# Paradoxical activation of AMPK by glucose drives selective EP300 activity in colorectal cancer

**DOI:** 10.1371/journal.pbio.3000732

**Published:** 2020-06-30

**Authors:** María Gutiérrez-Salmerón, José Manuel García-Martínez, Javier Martínez-Useros, María Jesús Fernández-Aceñero, Benoit Viollet, Severine Olivier, Jagat Chauhan, Silvia R. Lucena, Antonio De la Vieja, Colin R. Goding, Ana Chocarro-Calvo, Custodia García-Jiménez

**Affiliations:** 1 Area of Physiology, Faculty of Health Sciences, University Rey Juan Carlos, Alcorcón, Madrid, Spain; 2 Translational Oncology Division, OncoHealth Institute, Health Research Institute-University Hospital Fundación Jiménez Diaz-UAM, Madrid, Spain; 3 Department of Surgical Pathology, Hospital Gregorio Marañon, Madrid, Spain; 4 Université de Paris, Institut Cochin, CNRS, INSERM, Paris, France; 5 Ludwig Institute for Cancer Research, Nuffield Department of Medicine, University of Oxford, Oxford, United Kingdom; 6 Unidad de Tumores Endocrinos (UFIEC), Instituto de Salud Carlos III and CiberOnc, Majadahonda, Madrid, Spain; Duke University, UNITED STATES

## Abstract

Coordination of gene expression with nutrient availability supports proliferation and homeostasis and is shaped by protein acetylation. Yet how physiological/pathological signals link acetylation to specific gene expression programs and whether such responses are cell-type–specific is unclear. AMP-activated protein kinase (AMPK) is a key energy sensor, activated by glucose limitation to resolve nutrient supply–demand imbalances, critical for diabetes and cancer. Unexpectedly, we show here that, in gastrointestinal cancer cells, glucose activates AMPK to selectively induce EP300, but not CREB-binding protein (CBP). Consequently, EP300 is redirected away from nuclear receptors that promote differentiation towards β-catenin, a driver of proliferation and colorectal tumorigenesis. Importantly, blocking glycogen synthesis permits reactive oxygen species (ROS) accumulation and AMPK activation in response to glucose in previously nonresponsive cells. Notably, glycogen content and activity of the ROS/AMPK/EP300/β-catenin axis are opposite in healthy versus tumor sections. Glycogen content reduction from healthy to tumor tissue may explain AMPK switching from tumor suppressor to activator during tumor evolution.

## Abstract 2

Metabolic context determines whether the key energy sensor AMPK is a tumor suppressor or tumor promoter. This paradoxical behavior is explained through glucose inhibition of AMPK in healthy tissue versus glucose induction of AMPK in cancer colon epithelial cells.

## Introduction

Coordination of gene expression programs that drive proliferation and differentiation is essential to maintain homeostasis and for development. Understanding how the availability of nutrients needed for macromolecule synthesis is coordinated with gene expression reprogramming is a key issue. Recent insights have revealed a key integrating role for the evolutionarily conserved lysine acetyl-transferases EP300 and the highly related CREB-binding protein (CBP), which target histone and nonhistone proteins to modify their interactions and/or activities [1]. Best known as co-factors for a wide range of transcription factors—including nuclear receptors, β-catenin, Smad, or p53—EP300 and CBP share most targets, and as functional differences remain unclear, they are often referred to as EP300/CBP. In addition to acting as transcription co-factors, EP300/CBP have also been implicated in DNA damage repair and control of proliferation [2], with increasing evidence suggesting a role in regulating metabolism [3–5] and autophagy [6,7]. Given the broad repertoire of interactors and the limited pools of EP300/CBP, it has been suggested that there may be competition for EP300/CBP [1], raising the possibility that environmental cues signal to direct EP300/CBP interactions towards specific pathways. For example, recruitment of EP300/CBP to the cAMP-response element binding protein CREB is enhanced by CREB phosphorylation in response to elevated cAMP [8] or intracellular calcium [9]. Alternatively, direct post-translational modification (PTM) of EP300/CBP itself may regulate its activity or association with specific interactors.

Several intra- and extracellular inputs modulate EP300/CBP activity, with PTMs such as methylation, ubiquitination, sumoylation, or phosphorylation [10] altering protein stability, interactions, and ultimately function [11]. Most notable are phosphorylation events within the C-terminal (Carboxy terminus [Ct]) region that relieve inhibition of the acetyl-transferase domain through a conformational switch [7]. EP300 can be phosphorylated within the Ct by AKT [12] and the mitogen-activated protein kinase (MAPK) extracellular signal-regulated kinase (ERK) [10] downstream from pro-proliferative receptor tyrosine kinases as well as by mammalian Target Of Rapamycin Complex 1 (mTORC1) [7], which is in turn activated by amino acids needed for proliferation. In-trans auto-acetylation of EP300 was suggested to yield increased acetyl-transferase activity that may enhance its stability [13,14]. In addition to the Ct regulatory inputs, the amino terminus (Nt) of EP300 is also modified at S89 by protein kinase C (PKC) or AMP-activated protein kinase (AMPK), a key cellular sensor that controls the balance between catabolism and anabolism. AMPK-mediated phosphorylation of EP300 prevents its interaction with nuclear hormone receptors [15]. Since AMPK is generally activated in response to reduced glucose availability or by increased AMP:ATP or ADP:ATP ratio [16,17], AMPK-driven inhibition of EP300/nuclear receptor interaction provides a means to coordinate its activity with the supply of energy. However, it is unclear whether the response to changing glucose levels is specific to EP300 or CBP, whether it may affect gene expression programs independent of nuclear receptors, or whether the AMPK response differs between cell types. Especially relevant would be differences between cells that have physiological roles in glucose sensing and cells that exhibit metabolic pathologies such as cancer cells.

Given the critical importance of EP300/CBP for gene expression, understanding their regulation by the metabolic context during tumor evolution is a key issue. Here we explore the response of EP300 to changing glucose levels in gastrointestinal cancer cells. We show in vitro, in vivo, and in human clinical samples that, in contrast to expectation, exposure to glucose in colon cancer cells triggers AMPK activation that increases EP300 levels and redirects EP300 away from nuclear hormone receptors towards β-catenin, a key driver of colorectal cancer proliferation. By contrast, in healthy colon or other cancer cell types, glycogen storage blocks the ability of glucose to activate the reactive oxygen species (ROS)/AMPK/EP300/β-catenin axis.

## Results

### Cell-type–specific activation of EP300 but not CBP by glucose

**Fig 1:**
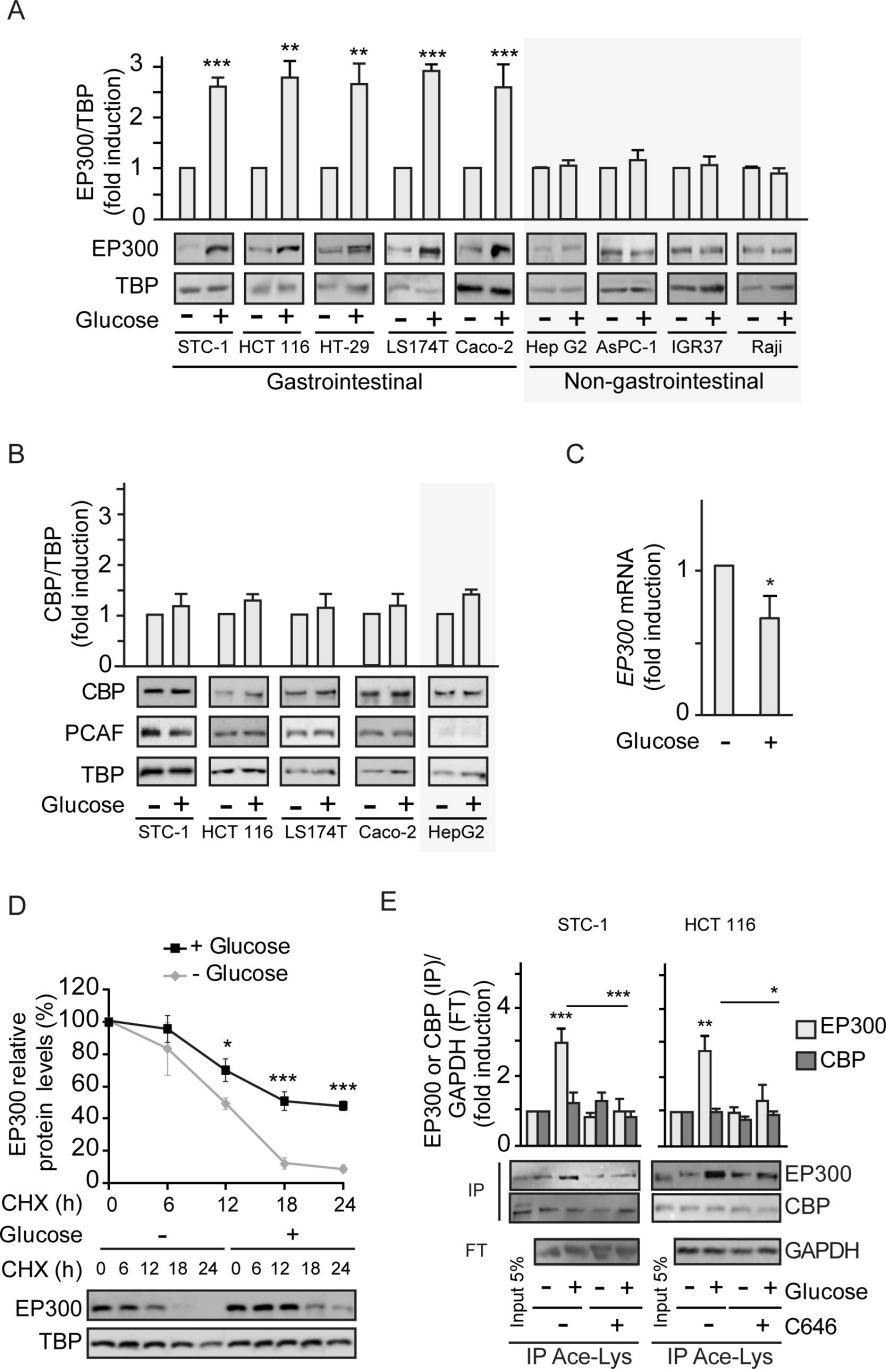
Glucose selectively increases EP300 in a cell-type–specific manner in gastrointestinal cancer cells. **See related results in** S1 **Fig** HCT 116 or the indicated cells were starved of glucose for 36 h (−) before addition of glucose 25 mM for 24 h (+). Where indicated, the EP300 inhibitor C646 was added at 5 μM for the last 24 h. All panels except (C) contain representative western blots and statistical analysis of 3 independent experiments using whole cells extracts from indicated gastrointestinal or non-gastrointestinal cancer cells (A, B, and D) or extracts immunoprecipitated with anti-Ace-Lys antibody (E). (A) Comparison of EP300 induction by glucose in gastrointestinal and non-gastrointestinal cancer cell lines. (B) Selectivity of the cell-type–specific EP300 induction by glucose evaluating the close related CBP and associated PCAF acetyl transferases. (C) RT-qPCR of EP300 mRNA from HCT 116 cells starved of or cultured with glucose 25 mM for 24 h. Results show fold-induction normalized to 18S RNA expression. (D) Protein synthesis was inhibited with CHX and the degradation of EP300 was monitored in cells cultured without or with 25 mM glucose (time course) by western blotting of whole cell extracts. (E) Immunoprecipitation with anti-Ace-Lys antibody followed by western blotting using anti-EP300 or anti-CBP. GAPDH is the loading control shown in the FT. Input lane is not in the quantification. For statistical analysis, *n* ≥ 3 and values represent mean ± SEM; *P < 0.05; **P < 0.01; ***P < 0.001. (A–C) by Student *t* test and (D–E) by one-way ANOVA. Individual data can be found as S1 Data and underlying raw images at S1 Raw Images. Ace-Lys, acetyl-lysine; CBP, CREB-binding protein; CHX, cycloheximide; EP300, Histone acetyltransferase p300; FT, flow through; GAPDH, Glyceraldehyde 3-phosphate dehydrogenase; PCAF, P300/CBP-associated factor; RT-qPCR, quantitative Reverse Transcription Polymerase Chain Reaction; TBP, TATA-box-Binding Protein.

Glucose uptake can fuel glycolysis to generate acetyl-CoA, a key co-factor for the EP300/CBP acetyl transferase [18,19] that should be important for several pathologies, including diabetes and cancer [1]. Addition of glucose to gastrointestinal murine (STC-1) or human (HCT 116) cancer cells previously starved of glucose for 36 h leads to significantly increased cytoplasmic and nuclear EP300 levels as detected by western blotting (S1A Fig) and immunofluorescence (S1B Fig); note that EP300 major fraction is nuclear. Remarkably, glucose-mediated induction of EP300 levels is restricted to gastrointestinal cancer cells (Fig 1A) with no effects in cancer cells from associated organs like pancreas AsPC-1 or liver Hep G2, or unrelated human cancer cells like IGR37 melanoma or Raji B-cell lymphoma. Surprisingly, despite the high homology of EP300 and CBP and the lack of identified stimuli that discriminate between them, exposure to glucose selectively increased EP300, as shown in Fig 1A and S1A and S1B Fig, without altering the levels of CBP or the associated acetyltransferase P300/CBP-associated factor (PCAF) (Fig 1B).

Although we anticipated elevated EP300 levels in response to glucose to be mediated by increased EP300 transcription, as reported for human endothelial umbilical vein cells (HUVEC) [3], unexpectedly, EP300 mRNA levels were moderately decreased in response to glucose (Fig 1C), indicating that glucose might increase EP300 protein stability. Indeed, inhibition of protein synthesis by cycloheximide (CHX) revealed augmented EP300 protein stability in HCT 116 colon cancer cells exposed to glucose (Fig 1D), likely triggered by PTMs.

Since auto-acetylation of EP300/CBP in trans enhances its catalytic activity [13,14,20], EP300 acetylation status in response to glucose addition was examined by immunoprecipitation of cell extracts using an anti-acetyl lysine antibody followed by western blotting. The results revealed increased levels of acetylated EP300, but not acetylated CBP, following stimulation of STC-1 and HCT 116 cells with glucose (Fig 1E), with acetylation of EP300 being blocked using the selective EP300 inhibitor C646 [21]. Acetylation of H3K9, a specific target of EP300 but not of CBP [22,23], was also increased by glucose exposure and was prevented by C646 (S1C Fig).

Collectively, the results indicate that in gastrointestinal, but not in multiple other cancer cell types, glucose levels selectively increase EP300 but not CBP. The data suggest that additional high-glucose–induced PTMs in EP300 may mediate its pro-proliferative effects on colon cancer cells.

### Selective induction of EP300 by high glucose correlates with cell-type–specific AMPK activation

**Fig 2:**
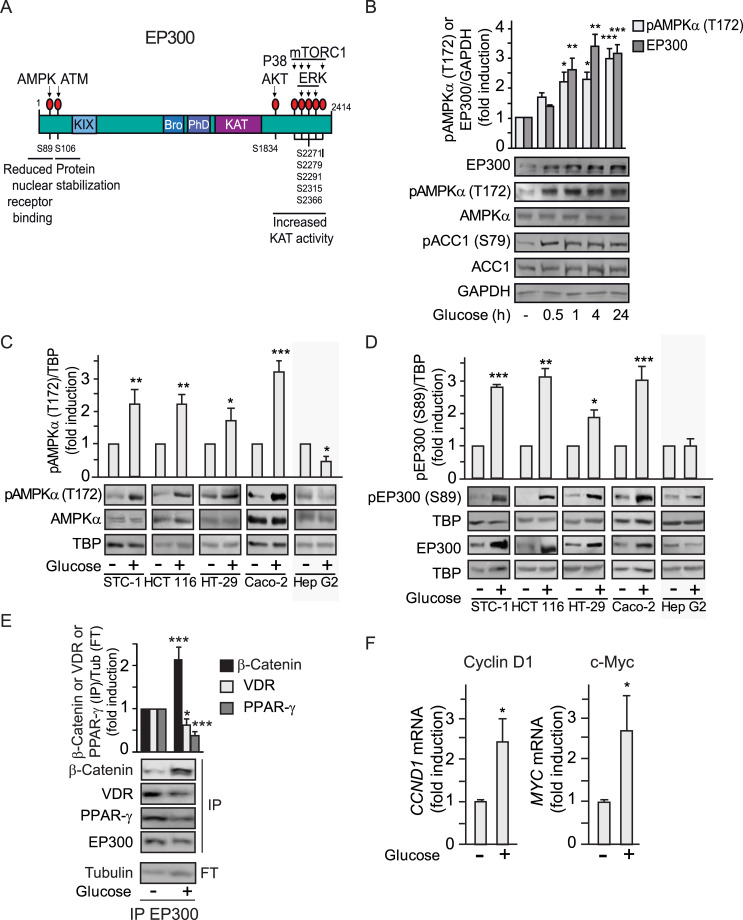
Glucose induction of EP300 correlates with cell-type–specific AMPK activation in gastrointestinal cancer cells. **See related results in** S2 **Fig**. (A) Scheme showing location of EP300 phosphorylation sites and responsible kinases. (B–D) Representative western blots of whole cell extracts from indicated cells cultured for 36 h in the absence of glucose (−) or in the presence of 25 mM glucose (+); GAPDH or TBP, loading controls. (C) Gastrointestinal cell-type–specific induction of pAMPK (T172). (B) Time course of pAMPK (T172) and EP300 induction upon addition of glucose 25 mM to STC-1 cells for the indicated times. The canonical AMPK substrate pACC1 (S79) is shown. (D) Gastrointestinal cell-type–specific induction of pEP300 (S89) as read out of pAMPK (T172) induction by glucose. (E) Immunoprecipitation with anti-EP300 antibody followed by western blotting using anti-β-catenin, anti-VDR, or anti-PPARγ. Tubulin, loading control shown in the FT. (F) RT-qPCR of the β-catenin target genes: CYCLIN D1 (*CCND*1) and *MYC* from HCT 116 cells. Values normalized with endogenous control (18S RNA) are refer to as fold induction over untreated cells. Statistical analysis of n ≥ 3 and values represent mean ± SEM; *P < 0.05; **P < 0.01; ***P < 0.001. (B) by one-way ANOVA, (C–F) by Student *t* test. Underlying data can be found as S1 Data and raw images at S1 Raw Images. AKT, Serine-Threonine Kinase AKT or PKB; AMPK, AMP-activated protein kinase; ATM, Serine-protein kinase ATM (Ataxia telangiectasia mutated); Bro, Bromodomain; ERK, extracellular signal-regulated kinase 1; FT, flow through; GAPDH, Glyceraldehyde 3-phosphate dehydrogenase; KAT, Lysine acetyl transferase catalytic center; KIX, Nuclear receptor and CREB interacting domains; mTORC1, mammalian Target Of Rapamycin Complex 1; PhD, PhD finger; PPARγ, Peroxisome Proliferator-Activated Receptor gamma; P38, Mitogen-activated protein kinase P38; RT-qPCR, quantitative Reverse Transcription Polymerase Chain Reaction; TBP, TATA-box-Binding Protein; VDR, vitamin D receptor.

We reasoned that differential interactions and enhanced acetylation of EP300 molecules in response to glucose may rely on additional PTMs and initially explored the possibility that glucose would regulate one of the several kinases known to phosphorylate EP300 (Fig 2A). Using H_2_O_2_ as a positive control known to induce ERK, p38, AKT [24,25], and AMPK [26], glucose only activated AMPK among these kinases (S2A Fig). We therefore focused our attention on AMPK, a kinase that we anticipated would be inhibited by glucose addition. To our surprise, time course experiments in gastrointestinal cancer cells revealed that glucose induced a significant activation of AMPK, as detected by the activating phosphorylation on T172, as well as increased phosphorylation of the canonical AMPK substrate Acetyl-Coenzyme A Carboxilase 1 (ACC1) on S79 (Fig 2B). Most notably, like EP300, AMPK was induced by glucose in the panel of gastrointestinal cancer cell lines, but not in hepatocellular carcinoma Hep G2 cells (Fig 2C). Importantly, phosphorylation at the EP300 consensus AMPK site (S89) correlated with increased EP300 protein levels in gastrointestinal cancer cells but not in Hep G2 liver cancer cells (Fig 2D).

Elevated EP300 levels by glucose may generally increase EP300-substrate interactions, leading to increased acetylation of EP300 substrates: EP300 itself and H3K9, as shown in Fig 1E and S1C Fig. However, since phosphorylation of EP300 (S89) by AMPK was previously reported to decrease EP300/Peroxisome Proliferator-Activated Receptor gamma (PPARγ) interactions in BHK cells [15], co-immunoprecipitations with anti-EP300 antibodies were used to evaluate EP300 interactions. EP300/PPARγ interactions, examined as a control, were down-regulated also in gastrointestinal cancer cells (Fig 2E). Likewise, EP300 interactions with the nuclear receptor for vitamin D (VDR), associated with colon differentiation, were also decreased, extending previous findings. Surprisingly, by contrast, the interactions of EP300 with the pro-proliferative factor β-catenin, the effector of Wnt signaling critical for colon tumorigenesis, were remarkably increased. Furthermore, glucose induction of EP300/β-catenin interaction correlated with activation of β-catenin target genes critical for proliferation such as *CCND1* (cyclin D) and *MYC* as revealed by quantitative Reverse Transcription Polymerase Chain Reaction (qRT-PCR) (Fig 2F).

In sum, the results indicate that glucose promotes a selective switch in EP300 to favor proliferation-related EP300/β-catenin interactions and suppresses differentiation-related EP300/VDR interactions.

### Cell-type–specific induction of EP300 by glucose relies on AMPK activation

**Fig 3:**
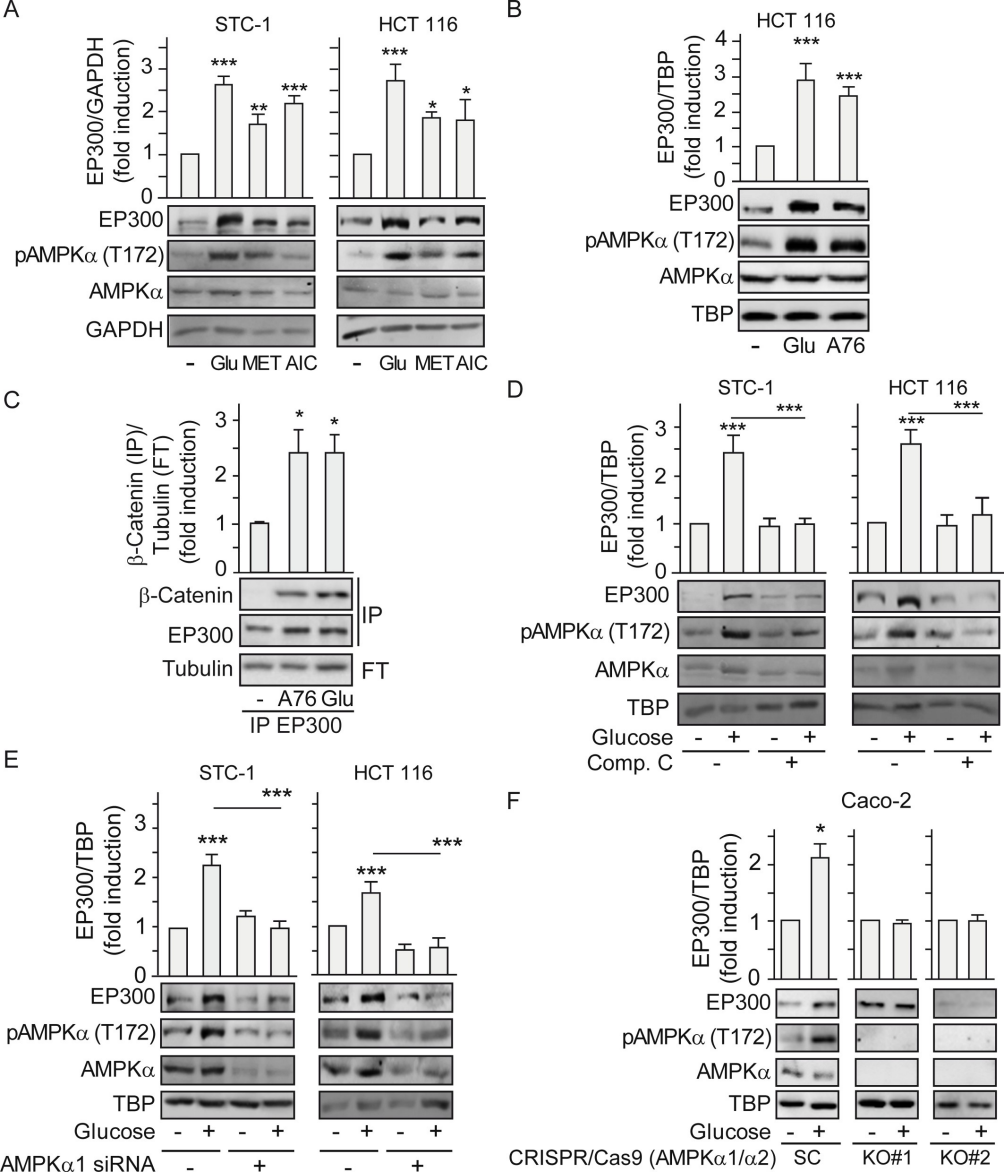
Glucose induction of EP300 and its interactions with β-catenin rely on AMPK activation. **See related results in** S3 **Fig**. Indicated cells were cultured for 36 h in the absence of glucose (−) or in the presence of 25 mM glucose (+) for 24 h. All treatments with inducers or inhibitors as indicated were for 24 h. Representative western blots and statistical analysis of whole cell (A–B) and (D–F) or immunoprecipitated extracts with anti-EP300 antibody (C). GAPDH, TBP, or Tubulin are loading controls. (A) Effect of MET 5 mM and AICAR (AIC) 20 μM compared to glucose 25 mM. (B) Effect of the specific AMPK inducer A-769662 (A76) 25 μM on EP300 induction. (C) Effect of AMPK induction by A-769662 (A76) 50 μM on the EP300/β-catenin interactions. (D) Effect of AMPK inhibition by Compound C (20 μM) on EP300 induction. (E) Depletion of the catalytic subunit of AMPK by transfection with AMPKα-specific siRNA (or an SC) for 48 h. Indicated cells were starved of glucose 24 h and refed with 25 mM glucose for another 24 h. (F) Effect of deletion of AMPKα on EP300 induction by glucose in colon cancer cells. Caco-2 colon cancer cells CRISPR/Cas9 deleted of AMPK α1 and α2 (AMPKα1/α2 KO) are compared to the corresponding SC [29]. Statistical analysis by one-way ANOVA (A–E) or by Student *t* test (F). Values represent mean ± SEM; *n* ≥ 3; *P < 0.05; **P < 0.01; ***P < 0.001. Individual data can be found as S1 Data and underlying raw images at S1 Raw Images. AICAR, 5-aminoimidazole-4-carboxamide ribonucleotide; AMPK, AMP-activated protein kinase; A76, AMPK inducer A-769662; Comp. C, Compound C; GAPDH, Glyceraldehyde 3-phosphate dehydrogenase; EP300, Histone acetyltransferase p300; KO, knockout; MET, metformin; SC, scramble control; TBP, TATA-box-Binding Protein.

The ability of glucose to stimulate AMPK activity in gastrointestinal cancer cells suggested that it was a candidate regulator of the cell-type–specific response of EP300 to glucose. To determine whether EP300 protein levels increased in response to glucose through AMPK induction, we manipulated AMPK activity and examined the EP300 response. Cells were exposed to metformin or 5-aminoimidazole-4-carboxamide ribonucleotide (AICAR) that pharmacologically activate AMPK indirectly [27], with glucose being used as a positive control. Fig 3A reveals that both metformin and AICAR increased pAMPK (T172) and induced a parallel increase in EP300 levels in STC-1 and HCT 116 cells. Likewise, direct induction of AMPK with the activator A769662, which binds to the AMPK beta1 subunit and acts both allosterically and via inhibition of AMPK (T172) dephosphorylation [28,29], also increased EP300 levels in HCT 116 (Fig 3B). Importantly, direct induction of AMPK with A769662 led to enhanced interaction between EP300 and β-catenin (Fig 3C), that can promote colon cancer proliferation during tumorigenesis. Conversely, inhibiting AMPK using the ATP competitive Compound C blocked glucose induction of pAMPK (T172) and of EP300 (Fig 3D). Small interfering RNA (siRNA) mediated depletion of AMPK, and consequently of pAMPK (T172), also blocked EP300 accumulation in response to glucose in STC-1 and HTC 116 gastrointestinal cancer cell lines (Fig 3E). To eliminate any potential off-target effects of the siRNA or pharmacological regulators of AMPK activity, we obtained 2 different clones of CRISPR/Cas9-mediated AMPK α1 and α2 knockout (KO) Caco-2 colon cancer cells [30,31]. Although the parental scramble Caco-2 cells responded to glucose by inducing EP300 protein levels, this effect was ablated in the AMPKα KO cells (Fig 3F). Furthermore, expression of a constitutively active deletion mutant of AMPK [32] reproduced the accumulation of EP300 in response to glucose (S3A Fig). By contrast, neither inhibition of EP300 using C646 (S3B Fig) nor overexpression of EP300 (S3C Fig) altered the induction of AMPK phosphorylation by glucose, indicating that EP300 lies downstream of AMPK and not upstream. Collectively, the data so far are consistent with glucose-driven activation of AMPK in gastrointestinal cancer cells leading to increased EP300 protein levels and selective interactions and activity.

### Glucose metabolism-generated ROS activate AMPK in cancer cells that cannot store glycogen

Since AMPK is a known energy sensor induced by starvation, it was surprising that the mechanism by which glucose induced EP300 in gastrointestinal cancer cells was through AMPK activation. AMPK can be stimulated through both canonical and noncanonical pathways. Canonical, energy-sensing pathways are dependent on increased intracellular AMP/ATP, ADP/ATP ratios. Noncanonical pathways are dependent on stresses, such as increased intracellular Ca^+2^ or decreased fructose-1,6-bisphosphate (FBP levels) and are exemplified by ROS induction or glucose deprivation [33–37]. To explore the mechanism of AMPK activation by glucose, we examined whether, in gastrointestinal cancer cells, 25 mM glucose might induce osmotic stress that causes AMPK activation. STC-1 cells were cultured in parallel with similar concentrations of glucose or mannitol, a membrane-impermeable carbohydrate. Unlike glucose, mannitol induced accumulation neither of pAMPK (T172) nor of EP300 or EP300 phosphorylation (S89) (S4A Fig), indicating that osmotic stress is not the cause of EP300 induction. The use of 2-deoxy-glucose (2-DG), a non-metabolizable carbohydrate, allowed us to explore the need of glucose metabolism for AMPK activation in gastrointestinal cancer cells. While both pAMPK (T172) and EP300 levels were induced by glucose in HCT 116 cells, addition of 2-DG interfered with glucose-mediated activation of AMPK and EP300 (S4B Fig). Together, these results suggest that glucose metabolism is necessary for AMPK induction. In consequence, we hypothesized that perhaps in Hep G2 cells (in which glucose did not induce AMPK), glucose was not metabolized but was stored instead as glycogen.

**Fig 4:**
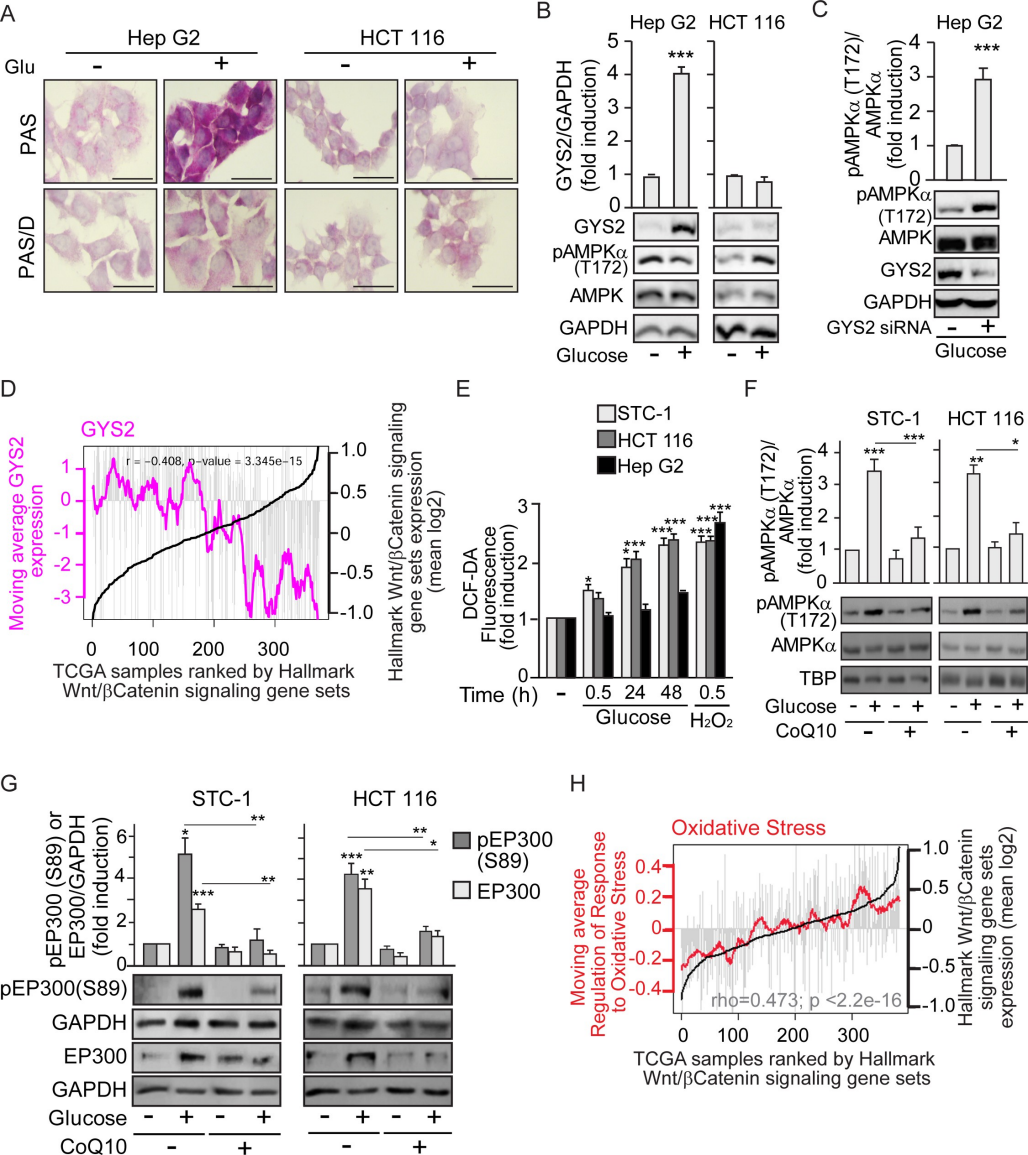
Cell-type–specific glucose induction of AMPK depends on the fate of glucose: To be stored as glycogen or metabolized to increase ROS. **See related results in** S4 **Fig**. Cells were cultured as earlier; starvation of glucose lasted 24 h for cells that required pre-treatment with CoQ10 (10 μM) or transfection. Before starvation, pre-treatment with CoQ10 was 12 h and transfection for 48 h. (A) Glycogen stores in Hep G2 and HCT 116 colon cancer cells, revealed by PAS staining. Diastase treatment (PAS-D) degrades glycogen and reveals specificity of staining; scale bars: 25 μm. (B) Differential induction of the rate-limiting enzyme for glycogen synthesis GYS2 in Hep G2 and HCT 116. Representative western blots and statistical analysis with GAPDH as loading control. (C) Induction of pAMPK (T172) by glucose in Hep G2 cells upon depletion of GYS2 by transfection with GYS2-specific siRNA (+) or the SC (−). (D) Bioinformatic analysis of the TCGA human liver cancer (HCC) cohort. Individual samples were ranked by expression of the GSEA “HALLMARK β-catenin signaling” gene set (black line). Grey bars indicate levels of expression in each individual tumor sample of GYS2. Pink line represents the moving average GYS2 across each 20 liver cancer samples. *P* value and correlation coefficient (rho) are indicated. (E) ROS production in gastrointestinal STC-1 and HCT 116 and in hepatoblastoma Hep G2 cancer cells, measured by flow cytometry using DCF-DA 0.5 μM as a label in response to glucose 25 mM or H_2_O_2_ 100 μM as positive control. (F) and (G) Western blotting analysis as in (B) with GAPDH or TBP as loading controls. (F) Effect of CoQ10 blockade of ROS on induction of pAMPK (T172) by glucose. (G) Effect of CoQ10 on induction of pEP300 (S89) by glucose. (H) Bioinformatic analysis of TCGA human colon cancer cohort, gene expression data. Individuals are ranked by the GSEA “HALLMARK β-catenin signaling” gene set (black line). Grey bars indicate levels of expression in each individual tumor sample of the GSEA “Regulation of the response to oxidative stress” gene set. Red line represents the moving average of the oxidative stress signature across each 20 colorectal cancer samples. *P* value and correlation coefficient (rho) are indicated. Statistical analysis by Student *t* test (B–C) or one-way ANOVA (E–G) of *n* ≥ 3 independent experiments. Values represent mean ± SEM; *P < 0.05; **P < 0.01; ***P < 0.001. Underlying data can be found as S1 Data and raw images at S1 Raw Images. AMPK, AMP-activated protein kinase; CoQ10, Coenzyme Q10; DCF-DA, 2’7’-dichlorodihydrofluorescein diacetate; GAPDH, Glyceraldehyde 3-phosphate dehydrogenase; GSEA, gene set enrichment analysis; GYS2, Glycogen Synthase 2; PAS, Periodic Acid-Schiff; PAS-D, Periodic Acid-Schiff with alpha amylase; ROS, reactive oxygen species; SC, scramble control; TBP, TATA-box-Binding Protein; TCGA, The Cancer Genome Atlas.

Glycogen storage was assayed using Periodic Acid-Schiff (PAS) staining, and pre-digestion with α-amylase or diastase (Periodic Acid-Schiff with alpha amylase [PAS-D]) that degrades glycogen was used to determine the specificity of PAS staining for glycogen. The results revealed that Hep G2 cells exhibited a basal level of glycogen and dramatically augmented glycogen stores when exposed to glucose (Fig 4A). By contrast, HCT 116 colon cancer cells did not accumulate glycogen in response to high glucose exposure. In line with these results, glucose increased the levels of the rate-limiting glycogen synthesis enzyme Glycogen Synthase 2 (GYS2) in Hep G2 cells, but not in HCT 116 (Fig 4B). Note that increased GYS2 inversely correlated with pAMPK (T172) in Hep G2 cells. Remarkably, siRNA-mediated GYS2 depletion led to AMPK induction by glucose in liver Hep G2 cancer cells (Fig 4C). Thus, blocking glycogen synthesis triggers AMPK activation by glucose in otherwise unresponsive cells.

As glucose-mediated activation of the AMPK/EP300 axis favors EP300/β-catenin association and would be inhibited in the presence of high GYS2, we anticipated that in liver cancer, in which GYS2 levels vary considerably, high GSY2 activity would correlate with reduced β-catenin activity. Remarkably, Fig 4D shows that in The Cancer Genome Atlas (TCGA) liver cancer (HCC) cohort, high expression of Wnt/β-catenin targets strongly anti-correlated with GYS2 expression. A Kaplan-Meier plot of disease-free survival versus GYS2 content in this cohort (S4C Fig) indicated that high GYS2 expression is associated with significantly higher survival up to 80 months.

Since glucose metabolism was required for AMPK induction in gastrointestinal cancer cells unable to store glycogen, we evaluated the possibility that glucose metabolism-generated ROS can noncanonically induce AMPK [37]. In gastrointestinal cancer STC-1 and HCT 116 cell lines, high glucose concentrations increased the production of ROS over time up to levels comparable to those induced by 30-min treatment with 100 μM H_2_O_2_ (Fig 4E and S4D Fig). By contrast, Hep G2 hepatocellular carcinoma cells, which exhibited lower basal ROS, did not significantly increase ROS production upon exposure to glucose (Fig 4E). Lack of ROS production together with increased glycogen content in Hep G2 upon glucose exposure (Fig 4A), inhibited pAMPK (T172) in these cells (S4E Fig), ERK 1/2 phosphorylation is shown as a positive control for H_2_O_2_ [38]. Significantly, siRNA-specific depletion of GYS2 in Hep G2 cells cultured with high glucose led to increased ROS (S4F Fig), consistent with glucose induction of pAMPK (T172) in Hep G2 depleted of GYS2 as shown in Fig 4C.

Coenzyme Q (CoQ10), or ubiquinone, acts as an electron carrier from complex I or complex II to complex III within the inner mitochondrial membrane and is also a potent antioxidant that neutralizes free radicals [39]. In gastrointestinal cancer cells, CoQ10 reduced the production of ROS in response to glucose or H_2_O_2_ (S4G Fig). Consistent with this, CoQ10 blocked the glucose-driven induction of pAMPK (T172) (Fig 4F) and the downstream pEP300 (S89) phosphorylation and accumulation of EP300 (Fig 4G). Importantly, glucose-mediated induction of H3K9 Ace was also prevented by CoQ10 (S4H Fig).

The results so far are consistent with a model in which colon cancer cells do not accumulate glycogen and glucose metabolism elevates ROS. Lack of glycogen and elevated ROS induce AMPK-mediated activation of EP300 to enhance β-catenin signaling. In line with this, a ROS-induced gene expression signature directly correlated with expression of a set of β-catenin target genes in the TCGA colorectal cancer cohort (Fig 4H). Relevant to these results, since glycogen synthase kinase 3β (GSK3β) induces β-catenin degradation and glycogen synthase inactivation, note that glucose did not significantly change GSK3β activity in colon cancer cells, according to unchanged levels of pGSK3β (S9) as previously published [40]. Thus, the inability of colorectal cancer cells to divert glucose away from metabolism into glycogen storage underpins the cell-type–specific activation of the glucose/ROS/AMPK/EP300/β-catenin axis.

### The ROS/AMPK/EP300/β-catenin axis drives glucose-mediated gastrointestinal cancer cell proliferation

Our results suggest that the interaction between EP300 and β-catenin, a key event in gastrointestinal neoplasia, is driven by glucose-mediated activation of AMPK via ROS. To confirm that this pathway drives proliferation, consistent with the ability of glucose to increase transcription of the β-catenin target genes *CCND1* and *MYC* (Fig 2F), we examined the effect of glucose and its downstream effectors on the cell cycle of gastrointestinal cancer cells. Flow cytometry indicated that increasing glucose levels accelerated cell cycle progression by shortening G1 to increase the number of cells in S and G2M (S5A Fig), with a total increase in the proliferation rate (S5B Fig).

**Fig 5:**
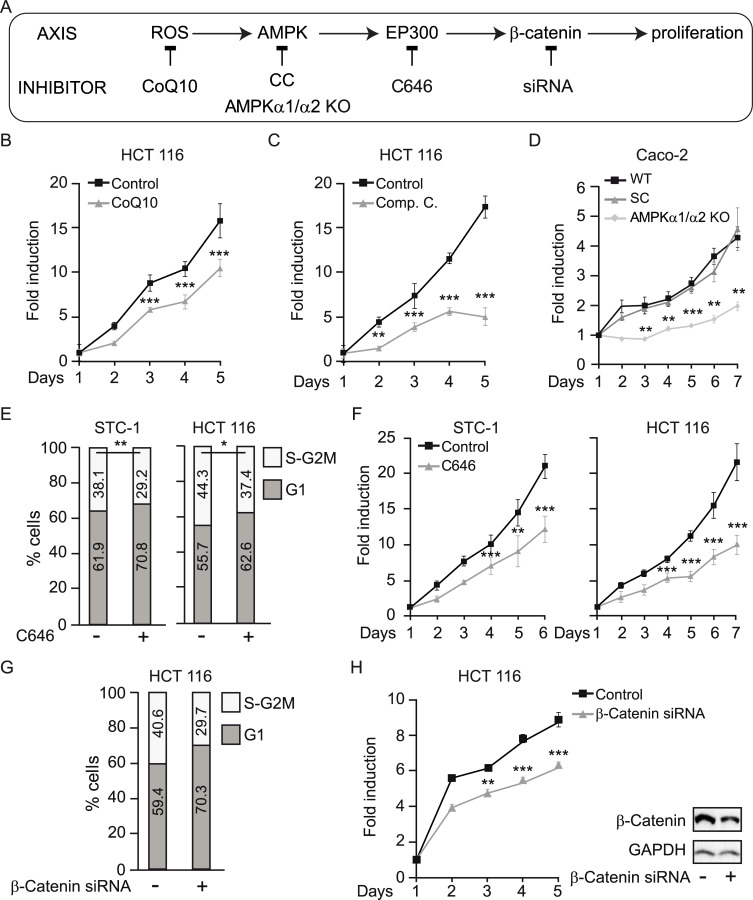
Glucose-induced proliferation of gastrointestinal cancer cells relies on ROS/AMPK/EP300/β-catenin signaling. **See related results in** S5 **Fig**. (A) Scheme of the axis evaluated with indicated blockade at each level: ROS were blocked by CoQ10; AMPK by Compound C or CRISPR/Cas9 deletion of α1 and α2 subunits; EP300 by C646 and β-catenin was depleted with a specific siRNA. (B–D), (F), and (H), proliferation curves; (E) and (G) flow cytometry analysis of cell cycle in indicated cancer cells cultured with 25 mM glucose under indicated conditions. (B) Effect on proliferation of ROS blockade by 10 μM CoQ10. (C) Effect on proliferation of AMPK inhibition with 20 μM Compound C. (D) Effect of CRISPR/Cas9-mediated deletion of AMPKα1 and α2. (E–F) Effect of the EP300 inhibitor C646 (5 μM) on the cell cycle (E) and on proliferation (F). (G–H) Effect of siRNA-mediated β-catenin depletion on cell cycle (G) and proliferation (H); western blots at the right of panel G are representative of the β-catenin depletion. Statistical analysis by Student *t* test; values represent mean ± SEM of *n* ≥ 3 experiments; *P < 0.05; **P < 0.01; ***P < 0.001. Individual data can be found as S1 Data and underlying raw images at S1 Raw Images. AMPK, AMP-activated protein kinase; AMPK α1/α2 KO, AMPK α1/α2 knock out; CC, Compound C; CoQ10, Coenzyme Q10; EP300, Histone acetyltransferase p300; ROS, reactive oxygen species; siRNA, small interfering RNA; WT, wild-type parental Caco-2 cells.

In support of a ROS/AMPK/EP300/β-catenin axis mediating glucose-induced proliferation of colon cancer cells, blockade at each level (Fig 5A) abolished the effects of glucose on proliferation. First, counteracting ROS accumulation with CoQ10 reduced the proliferative response of gastrointestinal cancer cells to high glucose (Fig 5B). Inhibition of AMPK with Compound C (Fig 5C) or AMPK deletion with CRISPR/Cas9 (Fig 5D) reduced proliferation to similar levels. Both AMPKα1/α2 KO clones (KO 1 and KO 2) were analyzed and gave similar results although, for simplicity, only clone KO 1 is shown. Moreover, inhibition of EP300 activity using C646 also interfered with high-glucose–induced acceleration of the cell cycle (Fig 5E) and decreased the cellular proliferation rate (Fig 5F). Finally, siRNA-mediated depletion of β-catenin also interfered with the acceleration of the cell cycle by glucose (Fig 5G) and blocked its pro-proliferative effects (Fig 5H), indicating that increased proliferation of colon cancer cells in response to glucose requires β-catenin.

Taken together, the results suggest that glucose specifically promotes gastrointestinal cancer cell growth through increased ROS production that induces AMPK and EP300-driven interaction with, and acetylation of, β-catenin. Acetylation of β-catenin is required for nuclear accumulation in colon cancer cells [40].

### The glucose/ROS/AMPK/EP300/β-catenin axis is on in mouse and human colorectal cancer

**Fig 6:**
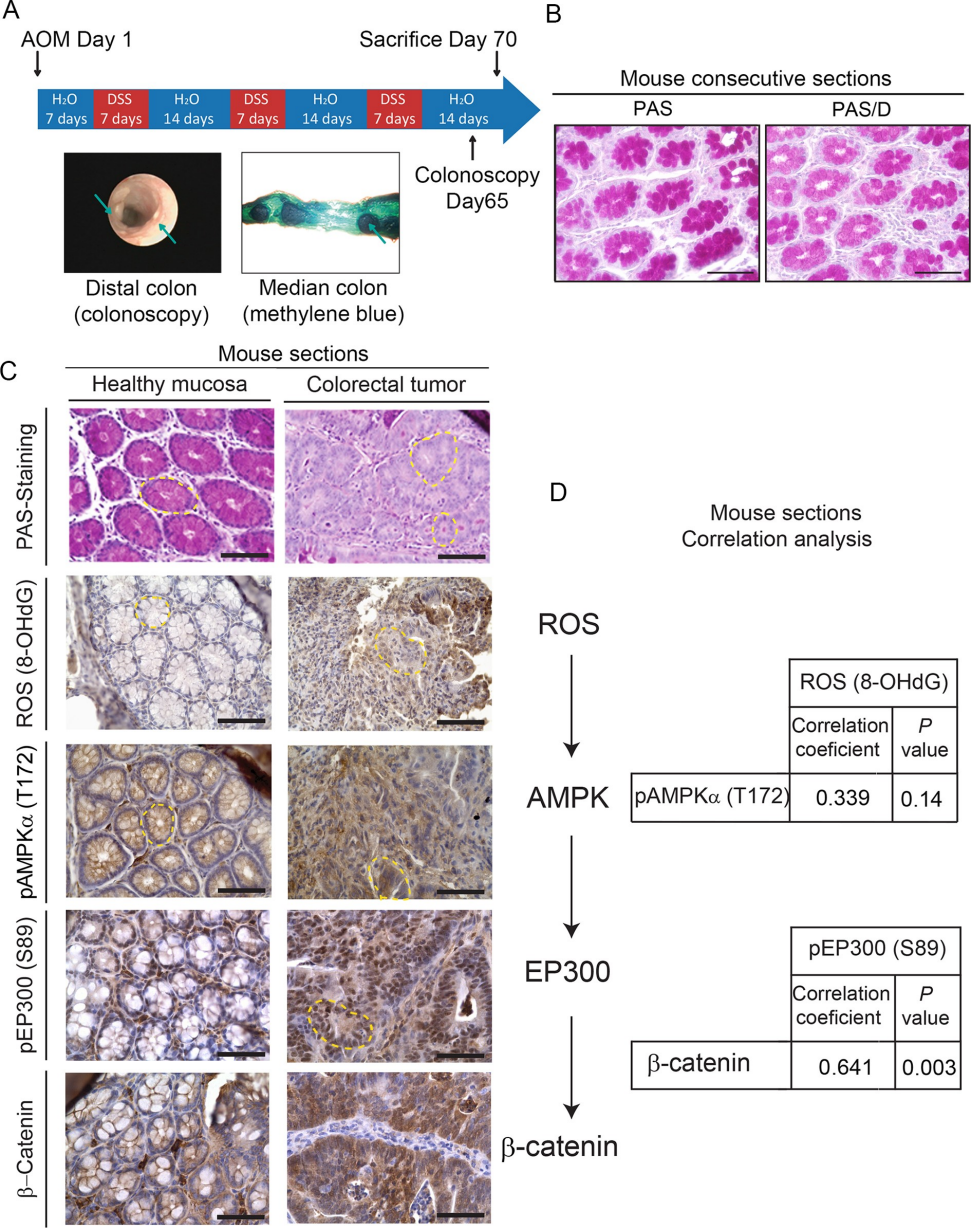
In vivo relevance of the ROS/AMPK/EP300/β-catenin axis in mouse colorectal cancer. (A) Scheme of the protocol to induce intestinal tumors in C57/Bl6 mice using AOM/DSS [42] with representative images of colonoscopy after the procedure and methylene blue staining of the medial portion of mouse intestine to show the tumors. (B) Representative pictures of histochemical analysis of glycogen content in healthy mouse intestinal mucosa by PAS staining preceded or not by diastase digestion (PAS/D) in consecutive sections to show glycogen specificity of PAS staining. Scale bars: 50 μm. (C) Analysis of glycogen content in relation with ROS (8-OHdG), pAMPK (T172), pEP300 (S89), and β-catenin. Representative photographs of mouse colorectal tumor or healthy intestinal mucosa stained with PAS or indicated antibodies (brown). Crypts are highlighted with yellow dashed line. Scale bars: 50 μm. (D) Correlation analysis of tumor and healthy sections from 19 mice with Pearson coefficient and *P* values shown. 8-OHdG, 8-hydroxy-2'-deoxyguanosine; AOM/DSS, azoxymethane/dextran sodium sulfate; pAMPKα (T172) phospho-AMP-activated protein kinase alpha (Threonine 172); pEP300 (S89), phospho-Histone acetyltransferase p300 (Serine 89); PAS, Periodic Acid-Schiff; PAS/D (or PAS-D), Periodic Acid-Schiff with diastase or alpha-amylase; ROS, reactive oxygen species.

To validate in vivo the pro-proliferative ROS-AMPK-EP300/β-catenin axis, we used combined azoxymethane and dextran sodium sulfate (AOM/DSS) to induce tumors in C57/Bl6 mice, a well-established model of colorectal cancer [41]. Fig 6A depicts the protocol to induce tumors with representative colonoscopy taken after tumor induction and methylene blue staining of the median intestinal section. PAS staining and pre-digestion with diastase (PAS-D) of consecutive sections revealed abundant glycogen content in healthy colon tissue (Fig 6B). In clear contrast with healthy tissue, tumor sections were PAS negative and thus depleted of any glycogen content (Fig 6C). In healthy tissue, glycogen stores anti-correlated with pAMPK (T172), pEP300 (S89), and nuclear β-catenin (Fig 6C). By contrast, tumor sections were depleted of glycogen and exhibited high levels of ROS, pAMPK (T172), pEP300 (S89), and nuclear and cytoplasmic β-catenin. Thus, tissue data are consistent with our results in cell lines. Immunohistochemistry from tumor and healthy sections from 19 mice were semi-quantitatively evaluated with an H-score, combining intensity of the staining and percentage of positive cells. The relationship between ROS (8-OHdG), pAMPK (T172), pEP300 (S89), and β-catenin was assessed by linear correlation (Fig 6D). pEP300 (S89) and β-catenin, factors located downstream of the axis, were positively correlated (R = 0.641; *P =* 0.003). Although linear correlation between the other factors did not achieve statistical significance, a high trend towards significance and a moderate positive correlation was found between ROS and pAMPK (T172) (R = 0.339; *P =* 0.144) and between pAMPK (T172) and β-catenin (R = 0.410; *P* = 0.072). Bearing in mind that the analysis was done in 19 isogenic mice, the results suggested that the ROS/AMPK/EP300/β-catenin axis might be “ON” in tumor tissue and “OFF” in adjacent healthy sections and encouraged us to study its relevance in human samples.

**Fig 7:**
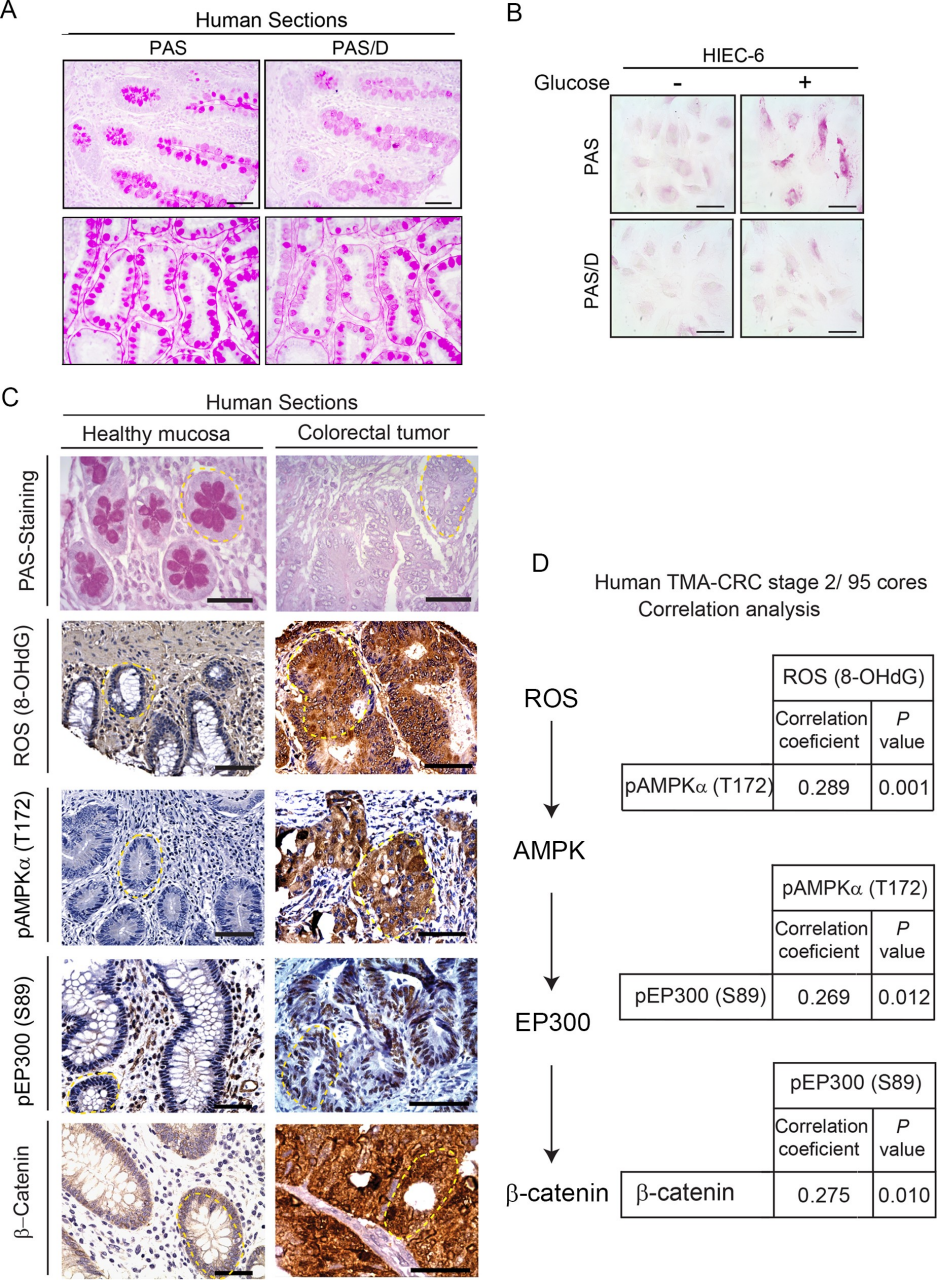
Clinical relevance of the ROS/AMPK/EP300/β-catenin axis in human colorectal cancer. (A) Histochemical staining with PAS preceded or not by digestion of consecutive sections with diastase (PAS/D) to reveal glycogen content (purple). Representative pictures with longitudinal (top) and transversal (bottom) sections. Scale bars: 50 μm. (B) Glycogen stores in human healthy colon cells HIEC 6 cultured for 24 h with low (1.5 mM) or high (25 mM) glucose revealed by PAS and PAS/D staining; scale bars: 25 μm. (C) Representative photographs of glycogen content stained with PAS in relation with ROS (8-OHdG), pAMPK (T172), pEP300 (S89), and β-catenin by immunohistochemical analysis in tumor versus healthy human bowel sections. Crypts are highlighted with yellow dashed line. Scale bars: 50 μm. (D) TMA analysis with 95 cores (0.6 mm diameter) of preselected regions of untreated CRC, stage 2. See Materials and Methods section and S1 Table for clinicopathological characteristics. Positive correlations were found between the nodes of the ROS/AMPK/EP300/β-catenin axis with correlation coefficients and *P* values shown. 8-OHdG, 8-hydroxy-2'-deoxyguanosine; CRC, colorectal cancer; pAMPKα (T172) phospho-AMP-activated protein kinase alpha (Threonine 172); PAS, Periodic Acid-Schiff; PAS/D, Periodic Acid-Schiff with alpha amylase; pEP300 (S89), phospho-Histone acetyltransferase p300 (Serine 89); ROS, reactive oxygen species; TMA, tissue microarray.

Consistent with the mouse results, PAS staining of healthy human tissue sections revealed the presence of abundant glycogen and neutral mucins, and pre-digestion of consecutive sections with diastase (PAS-D) confirmed the important contribution of glycogen (Fig 7A). The surprising capacity to store glycogen in healthy colon was confirmed in *in vitro*–cultured healthy colon HIEC 6 cells exposed to glucose 25 mM (Fig 7B).

The clinical relevance of the ROS/AMPK/EP300/β-catenin axis was examined in human samples (Fig 7C and 7D). Representative pictures of human large bowel sections (Fig 7C) illustrate that healthy sections were PAS positive in contrast with the PAS-negative tumor sections, as described for mouse. Moreover, the ROS/AMPK/EP300/β-catenin axis was active in human cancers and inactive in adjacent nontumor tissue, consistent with the in vivo data from mice. Core sections from 95 stage II colorectal cancer patients with the clinical features of tumors summarized in S1 Table were probed in tissue microarrays (TMAs) with the relevant antibodies. The immunohistochemistry from the TMA was semi-quantitatively evaluated with an H-score, combining intensity of the staining and percentage of positive cells. A linear correlation was assessed to study the relationship between ROS (8-OHdG), pAMPK (T172), pEP300 (S89), and β-catenin, (Fig 7D). A positive and significant linear correlation was revealed between each two nodes of the axis. First, ROS and pAMPK (T172) levels exhibited a positive and significant correlation (r = 0.289, *P =* 0.001). Second, a positive significant correlation (r = 0.269, *P* = 0.012) between pAMPK (T172) and pEP300 (S89) was found. Third, pEP300 (S89) and β-catenin were also directly correlated (r = 0.275, *P* = 0.010).

Taken together, the results suggest that the presence of glycogen in healthy colon epithelia at tumor initiation may inhibit AMPK, whereas upon tumor evolution, combined loss of capacity to store glycogen and increased glucose uptake and metabolism induce a ROS/AMPK/EP300/β-catenin axis that sustains proliferation of colon cancer cells.

## Discussion

The ability of cells to coordinate energy supply with gene expression is crucial to control migration, proliferation, and homeostasis [43]. It is widely assumed that the response to key nutrients such as glucose is likely to be similar between different cell types. In this respect, glucose limitation or starvation is known to activate AMPK in cells of diverse origins, including liver cancer Hep G2 cells [44,45]. However, most of these studies were done in tissues such as liver, muscle, and adipose that facilitate maintenance of whole-body energy homeostasis by releasing energy from glycogen or fat store. Remarkably, we reveal here that in gastrointestinal, but not in several other cancer cell types, AMPK is in fact activated by glucose. The apparently paradoxical activation of AMPK in gastrointestinal cancer cells arises as a consequence of ROS production generated by glucose metabolism and is suppressed in other cell types with an ability to synthesize and store glycogen. Thus, the fate of glucose within cells—increased metabolism and ROS accumulation versus storage as glycogen—is a key determinant for the response of AMPK, which in turn controls cellular metabolism and downstream transcription programs through effectors such as EP300.

Our results indicate that, in gastrointestinal cancer cells, the activation of AMPK in response to glucose leads to EP300 stabilization and increased interaction with β-catenin that can promote pro-proliferative *MYC* and *CCND1* expression. By contrast, AMPK-driven phosphorylation of EP300 interferes with EP300/VDR and PPARγ interactions, which are crucial for differentiation and homeostasis of gastrointestinal mucosa [46]. This is in line with previous observations that in BHK cells, phosphorylation of EP300 at S89 also decreases EP300 interactions with the nuclear receptors for thyroid hormones (TR) for All-trans Retinoic Acid (RAR) and for 9-cis Retinoic Acid (RXR) [15]. Our data are consistent with exposure to high glucose of gastrointestinal cancer cells, but not healthy or other cancer types, promoting a switch of a limited EP300 pool from nuclear receptors towards β-catenin leading to a pro-proliferative outcome. Since S89 is not conserved between EP300 and CBP, this may explain the specificity of EP300 regulation by glucose. It could be argued that phosphorylation of pEP300 at S89 is mediated by PKC as previously reported in in vitro assays or HeLa cells [47,48]. Although PKC and AMPK target similar consensus sequences, PKC exhibits additional requirements at position −3 that make EP300 a poor PKC substrate. Moreover, we have been unable to detect induction of PKC by high glucose in gastrointestinal cancer cells. Since the siRNA-mediated depletion or CRISPR/Cas9-mediated deletion of AMPK abolishes, and the constitutively active AMPK mutant mimics the induction of EP300 by glucose, we believe the major phosphorylation event on S89 is mediated by AMPK. Nevertheless, in some cell types, PKC may induce pEP300 (S89) indirectly through AMPK activation as shown in monocytes [49].

Unlike in gastrointestinal cancer cells, glucose triggers increased EP300 mRNA expression in endothelial HUVEC cells [3] and cardiomyocytes [50]. In the absence of EP300 phosphorylation at S89 to redirect EP300 towards β-catenin, increased EP300 levels may lead to a transcriptional outcome different from that observed in gastrointestinal cancer cells. Thus, our results illustrate how nutrients signal through AMPK to select EP300 binding partners and substrates that define the transcriptional outcome in a cell-type–specific fashion.

Since AMPK activation by high glucose in colon cancer cells drives proliferation, a question arises as to whether AMPK acts as a tumor promoter or as tumor suppressor. AMPK increases cellular glucose uptake [51–53], a cancer cell hallmark that would require AMPK activity. However, AMPK also inhibits mTORC1, the master controller of protein synthesis for growth, and as such AMPK would be expected to be inhibited in cancer cells. The importance of the question is reflected by the significant interest in targeting AMPK in cancer (https://clinicaltrials.gov). In fact, examples can be found where AMPK inhibition reduces growth in prostate cancer cells, whereas AMPK activation with metformin or phenformin reduces or delays formation of certain tumors [54,55], although it can be argued that the effects of metformin on cancer cells might be AMPK dependent or independent [56,57]. A recent in-depth review of genetic and animal evidence proposes that AMPK acts as a tumor suppressor at initial stages and as a tumor promoter in later stages of tumor evolution, when AMPK may become critical for metabolic adaptation and survival [58]. However, what in each cell or at a given time drives AMPK to act as tumor suppressor or promoter remains unclear. One possibility would be that exhausting the capacity to store glycogen, which can bind and negatively regulate AMPK [59–62], allows glucose-mediated generation of the AMPK activator ROS, switching AMPK from suppressor to promoter of cancer growth. This hypothesis is supported by our results showing the contrasting capacity to store glycogen in healthy and tumor cells of the colon. It is tempting to propose that at tumor initiation increased glucose uptake might still be diverted into glycogen storage to inhibit AMPK, enabling mTORC1 to allow growth and proliferation. During tumor evolution, the capacity to store glycogen is diminished, rewiring AMPK signaling to coordinate metabolism that allows survival. At this stage, glucose metabolism will increase ROS to induce AMPK and redirect EP300 towards pro-proliferative β-catenin interactions. Importantly, metformin reduces glycogen content in several cell types [63,64]. Following this reasoning, the role of AMPK targeting drugs may depend on tumor stage [65]. Thus, the metabolic context (i.e., capacity to store glycogen) may define whether glucose inhibits AMPK, acting as tumor suppressor at initial tumor stages, or induces AMPK acting as tumor promoter at advanced stages to drive EP300/β-catenin interactions. In this scenario, the potential for AMPK to act as tumor promoter or suppressor may depend on the capacity to store glycogen.

Thus, activation of AMPK by glucose in gastrointestinal cancer cells leads to a cascade of downstream events, including increased levels and selective acetyl transferase activity of EP300 on specific substrates such as histone H3K9 (a hallmark of chromatin opening) [66] and the WNT effector β-catenin, which increases its transcriptional activity [67], leading to increased proliferation. The surprising cell-type–restricted activation of EP300 and its re-direction towards β-catenin in response to glucose and AMPK signaling revealed here highlights a key molecular switch that integrates nutrient availability with regulation of β-catenin, a key driver of gastrointestinal tumorigenesis. Since hyperglycemia can trigger increased ROS and is a major cause of clinical complications associated with diabetes and obesity [68], the results presented have implications for those cancers associated with diabetes [69,70] and for the non–insulin-dependent response of cells to high glucose. The results may also have significant implications for understanding the response to drugs targeting AMPK, increasingly used in the treatment of both diabetes and cancer.

Future work looking closely at initial versus final stages of colorectal cancer will unveil the potential of strategies targeting glycogen [71] or the convenience of inducing or inhibiting AMPK in stratified populations.

## Materials and methods

### Ethics statement

Animal studies were approved by the ethics committee C2EA number 005 Charles Darwin, (number of approval: APAFIS#21551–2019071915156299 v4). All experiments complied with the EU guidelines for the protection of vertebra animals used for scientific purposes, under French authorization and approval number 75–886.

### Human samples

This TMA was part of a comprehensive study in which many prognostic factors were included and was reviewed and approved by the Institutional Review Board (IRB) of the Fundación Jimenez Diaz Hospital, which evaluated the study, granting approval on December 9, 2014, by the act number 17/14. Clinical samples were kindly supplied by the BioBank of Fundación Jiménez Díaz-Universidad Autónoma de Madrid (PT13/0010/0012). All patients gave written informed consent for the use of their biological samples for research purposes. Fundamental ethical principles and rights promoted by Spain (LOPD 15/1999) and the European Union EU (2000/C364/01) were followed. In addition, all patients’ data were processed according to the Declaration of Helsinki (last revision 2013) and Spanish National Biomedical Research Law (14/2007, of July 3).

Materials are listed in S2 Table.

### Cell culture

Mouse tumor enteroendocrine STC-1, colorectal adenocarcinoma (HT-29, HCT 116, LS 174T, Caco-2), and hepatocellular adenocarcinoma (Hep G2) were cultured in DMEM. Healthy colon cells (HIEC 6) were cultured in Opti-MEM. Pancreatic carcinoma (AsPC-1), melanoma (IGR 37), and Burkitt’s lymphoma (Raji) were cultured in RPMI. All media were supplemented with 10% fetal bovine serum, and cells grew at 37°C under 5% CO_2_. Cells starved of glucose for 24–36 h were stimulated as indicated. Caco-2 were deleted by CRISP/Cas9 of AMPK α1 and α2 using two different sgRNA guides that generated 2 clones or the scramble control (SC). These clones were generated as previously described [30,31] from parental Caco-2.

#### Transient transfections

For plasmid transfection, cells were seeded in plates at 50% confluence using JetPei PolyPlus reagent, following the manufacturer’s instructions. After 24 h, cells were cultured in absence of glucose and treated as indicated.

For AMPKα or GYS2 siRNA, cells plated in 6-well plates at 50% confluence were transfected using JetPRIME reagent following the manufacturer’s instructions. After 2 d, cells were cultured in the absence of glucose for 24 h, treated again with glucose for another 24 h, and collected to analyze by western blot.

For β-catenin siRNA, cells were plated in 24-well plates at 15% confluence and transfected using JetPRIME reagent following the manufacturer’s instructions. Cells were collected to analyze cell-growth curves, cell cycle or by western blot.

### Colorectal cancer induction using AOM/DSS

Eight- to ten-week-old C57BL/6 male mice were injected i.p. with 12.5 mg/kg AOM (A5486, Sigma-Aldrich) on day 1 of the protocol. Mice were exposed to 3 cycles of 1.5% DSS-containing (160110, MP Biomedicals) drinking water for 7 d with a switch to DSS-containing water at day 7, day 28, and day 49. Colonoscopy (TRICAM endoscope Karl Storz) was performed on anesthetized mice with inhaled isoflurane 65 d after AOM injection to evaluate tumor development (representative colonoscopy picture shown in Fig 6A). At day 70, tumor-bearing mice were euthanized by cervical dislocation to obtain nontumor and tumor tissues.

### Preparation of cell extracts

#### Whole cell extracts

Cells were washed with iced PBS before extract preparation and scraped in RIPA buffer (10 mM Tris HCl [pH 7.4], 5 mM EDTA, 5 mM EGTA, 1% Tryton X100, 10 mM Na_4_P_2_O_7_ [pH 7.4], 10 mM NaF, 130 mM NaCl, 0.1% SDS, 0.5% Na-deoxycholate). After 5 min on ice, cells were pelleted (12,000 rpm for 5 min, 4°C), and the supernatant was directly used as whole cell extract or frozen at −80°C.

#### Fractionated cell extracts

After washing as before, cells were scraped in hypotonic buffer (20 mM Hepes [pH 8.0], 10 mM KCl, 0.15 mM EDTA, 0.15 mM EGTA, 0.05% NP40 and protease inhibitors) and swollen on ice for 10 min before adding 1:2 volume of sucrose buffer (50 mM Hepes [pH 8.0], 0.25 mM EDTA, 10 mM KCl, 70% sucrose). Lysates were fractionated (5,000 rpm for 5 min at 4°C) to obtain the cytoplasmic fraction in the supernatant. Nuclear pellets were further washed twice with washing buffer (20 mM Hepes [pH 8.0], 50 mM NaCl, MgCl_2_ 1.5 mM, 0.25 mM EDTA, 0.15 mM EGTA, 25% glycerol and protease inhibitors), pelleted at 5,000 rpm, 5 min at 4°C, and resuspended in nuclear extraction buffer (20 mM Hepes [pH 8.0], 450 mM NaCl, MgCl_2_ 1.5 mM, 0.25 mM EDTA, 0.15 mM EGTA, 0.05% NP40, 25% glycerol and protease inhibitors) before centrifugation at 12,000 rpm for 5 min at 4°C to pellet and discard cell debris. The supernatants were used as nuclear fractions.

### Immunoprecipitation

Whole cell extracts were obtained in the same buffer without 0.1% SDS and 0.5% Na-deoxycholate. For immunoprecipitation from fractionated extracts, the hypotonic buffer was modified by adding 100 mM NaCl and 0.1% NP40. For immune complex formation, protein A/G-coated magnetic beads were washed 3 times with the extraction buffer before coating with the primary antibody for 2 h at 4°C in a rotating wheel, followed by 2 washes with the same buffer to eliminate unbound antibody, and then extracts were added O/N at 4°C in the rotating wheel. Immunocomplexes were washed twice and used for western blotting.

### Western blotting

Proteins from lysed cells or immunoprecipitates were denatured and loaded on sodium dodecyl sulfate polyacrylamide gels and transferred to polyvinylidene difluoride membranes. After blocking with 5% (w/v) BSA or milk, the membrane was incubated with the corresponding primary and secondary antibodies. The specific bands were analyzed using Thyphoon or ChemiDoc Imaging Systems.

### Immunofluorescence

Cells in cover slips were washed 3 times, fixed with 4% paraformaldehyde in PBS (pH 7.4) for 10 min, and washed again. Cells were permeabilized (PBS [pH 7.4], 0.5% Triton X-100, 0.2% BSA) for 5 min; blocked (PBS [pH 7.4], 0.05% Triton X-100, 5% BSA) for 1 h at room temperature; incubated with primary antibody over night at 4°C; and washed 3 times for 5 min and incubated with the secondary antibody for 1 h at room temperature. Slides were mounted, and images were acquired using a SP5 confocal microscope (Leica) with a 63× objective. Fluorescence intensity was quantified using Image J software. For each experiment, 3 different fields were evaluated per slide.

### PAS and PAS-D staining

HCT 116, HIEC 6 and Hep G2 cells seeded in coverslips were washed 3 times, fixed with 4% paraformaldehyde in PBS (pH 7.4) for 10 min, and washed again. For PAS-D staining, slides were incubated with 0.2% α-amylase (Diastase, Sigma-Aldrich) in PBS for 30 min at 37°C and rinsed in tap water and then with distilled water. For PAS staining, this step was performed with PBS. Slides were then oxidized for 5 min with 1% solution of periodic acid (Sigma-Aldrich), washed with tap water for 3 min, washed with distilled water for 1 min, and incubated in Schiff’s reagent (Sigma-Aldrich) for 20 min. Slides were washed first with distilled water for 5 s and then with tap water for 10 min. Counterstaining was performed with Hematoxylin Solution (Sigma-Aldrich) for 2 min and washed 3 times for 5 min with PBS. For tissue sections, slides were deparaffinized and hydrated before diastase digestion (1 h at 37°C) and/or PAS staining: 5 min in 1% solution of periodic acid followed by washes and incubation in Schiff’s reagent for 5 min. Slides were counterstained with Hematoxylin for 30 s. Images were acquired using a microscope (Zeiss) with a 40× objective for cultured cells or 20× for tissue sections.

### Measurement and imaging of ROS by fluorescence

Intracellular levels of ROS were determined using 2’7’-dichlorodihydrofluorescein diacetate (DCF-DA) in conjunction with cytometer and fluorescent microscopy. For cytometry, cells were harvested, washed with PBS, and loaded with 0.5 μM DCF-DA in the dark for 15 min at 37°C. The excess dye was flushed off, and cells were resuspended in PBS. Fluorescence intensity was quantified using FACSCalibur (Becton-Dickinson). For microscopy imaging, cells were treated with 10 μM DCF-DA for 30 min at 37°C, washed twice with PBS, and analyzed in a fluorescence microscope.

### Cell-growth curves and cell cycle

Cells were seeded at a density of 20,000 cells per well in a Corning 12-well plate and counted in a Neubauer camera by TB dye exclusion. Cell number was counted every 24 h for 5–7 d, and the medium was replaced every 3 d. For cell cycle analysis, cells were harvested by trypsinization, washed with PBS, fixed in 70% ethanol, stained with 7-AAD (Santa Cruz Biotechnology) for 10 min at 37°C, and analyzed by flow cytometry (FACSCalibur, Becton-Dickinson). The percentages of cells in the cell cycle phases were analyzed using CXP software (Becton-Dickinson).

### Gene expression analysis

Total RNA was isolated with TRIzol (Invitrogen). cDNA was generated from 1 μg of RNA following the manufacturer’s protocol. Reagents and detection systems were from Life Technologies. 18S ribosomal RNA primers served as a nonregulated control. Relative expression was calculated using the Ct method, expressed as 2−^ΔΔCt^ [72]. The PCR efficiency was approximately 100%.

### Evaluation of protein degradation

Protein degradation was evaluated after protein synthesis inhibition by the administration of 30 μg/mL of CHX to HCT 116 cells incubated 24 h before without or with 25 mM glucose. A time course was performed by western blot analysis of whole cell extracts.

### Bioinformatics analysis

Gene expression (RNA-seq) data from the TCGA colorectal and liver (HCC) cancer cohort was downloaded from the cBioportal for Cancer Genomics (http://www.cbioportal.org) using CGDS-R [73,74] following TCGA guidelines (http://cancergenome.nih.gov/publications/publicationguidelines). Individual gene expression values for the genes of interest as normalized RNA-Seq by Expectation Maximization (RSEM) read counts pre-processed through the TCGA/cBioportal projects. RSEM values less than 1 were set to 1 to avoid negative expression values upon log2-tranformation if necessary. The “Regulation of Response to Oxidative Stress” and “HALLMARK_WNT_BETA_CATENIN_SIGNALING” β-catenin gene expression signatures were obtained from the Molecular Signatures Database (MSigDB) (http://www.broadinstitute.org/msigdb) [75]. All colorectal or HCC cancer samples were ordered by increasing expression values of the average expression of the HALLMARK_WNT_BETA_CATENIN_SIGNALING gene set. The moving average expression of the “Regulation of Response to Oxidative Stress” gene set or GYS2 expression was calculated using a sample window size of *n* = 20, and trendlines were added to the barplots. An R-script for calculating and generating moving average plots of TCGA cancer cohorts implementing TCGA access via cBioportal has been described previously [76]. An asymptotic Spearman correlation test using original log2 expression values, not the moving average, was used to determine the significance of the Spearman rank correlation. Analysis of TCGA data by Kaplan Meier plotter available at http://kmplot.com/analysis/index.php?p=background [77].

### Patient samples

A total of 95 patients diagnosed with stage II colorectal cancer who underwent surgery at General and Digestive Tract Surgery Department, Fundación Jimenez Diaz University Hospital, Madrid, Spain, were assessed for eligibility. Clinicopathological characteristics of patients are summarized in S1 Table.

### TMA, immunohistochemistry, and quantification

#### Human samples

A TMA was constructed using the MTA-1 tissue arrayer (Beecher Instruments, Sun Prairie) for immunohistochemistry analysis and contained 95 cores. Each core (diameter 0.6 mm) was punched from pre-selected tumor regions in paraffin-embedded tissues. We chose central areas from the tumor, avoiding foci of necrosis. Staining was conducted in 2-μm sections. Slides were deparaffinized by incubation at 60°C for 10 min and incubated with PT-Link (Dako, Agilent) for 20 min at 95°C in low pH to detect p-AMPK (T172) antigen, or high pH buffered solution to detect 8-OHdG and pEP300 (S89) antigens. To block endogenous peroxidase, holders were incubated with peroxidase blocking reagent (Dako, Agilent) and then with the following dilutions of antibodies: 1:250 of anti-8-OHdG to detect ROS [78,79] 1:100 of anti-phospho-EP300 (S89) for 20 min or overnight incubation with a 1:50 dilution of anti-pAMPK (T172). All previously described antibodies presented high specificity. After that, slides were incubated for 20 min with the appropriate anti-Ig horseradish peroxidase-conjugated polymer (EnVision, Dako, Agilent) or, in the case of 8-OHdG, with 1:200 anti-goat-HRP (Bethyl Labs) to detect antigen-antibody reaction. Sections were then visualized with 3,3'-diaminobenzidine (Dako, Agilent) as a chromogen for 5 min and counterstained with Harrys’ Hematoxylin (Sigma Aldrich, Merck). Photographs were taken with a stereo microscope (Leica DMi1).

According to the human protein atlas (available at http://www.proteinatlas.org), a human intestinal tissue was used as a positive control for immunohistochemical staining to determine anti-phospho-EP300 (S89) concentration, a human kidney tissue for anti-pAMPK (T172) and human brain tissue for 8-OHdG.

Immunoreactivity of tumor sample was quantified blind with an Histoscore (H score) that considers both the intensity and percentage of cells stained for each intensity (low, medium, or high) following this algorithm (range 0–300): H score = (low%) × 1 + (medium%) × 2 + (high%) × 3.

Quantification for each patient biopsy was calculated blindly by 2 investigators (MJFA and JMU). pEP300 (S89) showed nuclear staining, pAMPK (T172) and 8-OHdG were mainly cytoplasmic, and β-catenin was in the nucleus and cytoplasm.

#### Mouse samples

Whole intestines were prepared as Swiss-rolls, paraffin embedded, and sections (5 μm) were stained/counterstained with PAS/hematoxylin to detect the presence of glycogen and with the relevant antibodies as for human samples.

### Statistical analysis

Results are presented as fold induction, mean ± SEM from 3 biological replicates. Tests for significance between 2 sample groups were performed with Student *t* test and ANOVA with Bonferroni’s post-test for multiple comparisons. Differences were considered statistically significant if *P* ≤ 0.05.

For immunohistochemical expression, the Kolmogorov-Smirnov test was used to determine whether calculated H scores for each of the antigens were well-modelled by a normal distribution. In our series, only pEP300 (S89) revealed normal distribution. Linear correlation between parametric variables (pEP300 [S89]) was evaluated by the Pearson test and nonparametric variables by Spearman’s test. pEP300 (S89) H_score was categorized as low or high expression levels using the median as the cut-off point since it showed a normal distribution.

## Supporting information

S1 Fig Glucose-induced EP300 correlates with increased Ace-H3K9 and pro-proliferative β-catenin target gene expression in CRC cells. **Related to Fig 1.** Cells were starved of glucose for 36 h (−) before addition of 5 mM or 25 mM glucose for 24 h (A) or glucose 25 mM for 24 h (+) (B–D). C646 (5 μM) was added for 24 h where indicated. (A) Representative western blots of EP300 and statistical analysis of cytoplasmic (CE) or nuclear (NE) extracts from indicated cell lines. GAPDH or TBP are loading controls for cytoplasmic and nuclear fractions, respectively. (B) Confocal immunofluorescence images of STC-1 cells using indicated antibodies (scale bars represent 25 μm) and quantification of fluorescence intensity using ImageJ software (lower panel); for each experiment, 3 different fields were evaluated per slide. (C) Representative western blot and statistical analysis of the correlation between glucose induction of EP300 and H3K9 acetylation in gastrointestinal cancer cell lines. The selective EP300 inhibitor C646 abolishes EP300 and H3K9 acetylation. Statistical analysis by one-way ANOVA (A) and (C) or Student *t* test (B); *n* ≥ 3; *P < 0.05, **P < 0.01; ***P < 0.001. See individual data at S1 Data and underlying raw images at S1 Raw Images. CE, cytoplasmic extracts; CRC, colorectal cancer; EP300, Histone acetyltransferase p300; GAPDH, Glyceraldehyde 3-phosphate dehydrogenase; H3K9 Ace, Histone H3 Lysine 9 acetylated; NE, Nuclear extracts; TBP, TATA-box-Binding Protein.
(TIF)

S2 Fig Glucose selectively induces pAMPK (T172) in gastrointestinal cancer cells. **Related to Fig 2.** (A) Kinase induction was analyzed in STC-1 whole cell extracts; H_2_O_2_ (100 μM)_,_ was used as positive control for induction of pERK, pAKT, pp38, and pAMPK activation. GAPDH, loading control. Kinases previously reported to modify EP300 were studied. AKT, Serine-Threonine Kinase AKT or PKB; AMPK, AMP-activated protein kinase; ERK, ERK, extracellular signal-regulated kinase 1; GAPDH, Glyceraldehyde 3-phosphate dehydrogenase; P38, Mitogen-activated protein kinase P38
(TIF)

S3 Fig A constitutively active AMPK mutant induces EP300; EP300 is downstream of AMPK. **Related to Fig 3.** (A) Whole cell extracts of STC-1 cells transfected with a Myc-tagged deletion mutant of AMPK catalytic subunit that is constitutively active (CA) for 48 h and then starved of, or treated with, glucose (25 mM) for 24 h. Note the molecular weight of the myc-AMPKα1-CA is 37 KDa versus 63 KDa of the full length since it contains only amino acids 1–312 [32]. (B) The EP300 inhibitor C646 (5 μM) was added to STC-1 or HCT 116 cells cultured as previously described for the last 24 h. C646 inhibition did not abolish AMPK induction by glucose. (C) HCT 116 cells transfected with control or pCDNA3-Flag-EP300 expression vector were cultured as previously described to analyze whether EP300 alters glucose induction of AMPK. Statistical analysis (B–C) by one-way ANOVA; *n* ≥ 3; *P < 0.05, **P < 0.01; ***P < 0.001. Individual data can be found as S1 Data and underlying raw images at S1 Raw Images. AMPK, AMP-activated protein kinase; GAPDH, Glyceraldehyde 3-phosphate dehydrogenase; EP300, Histone acetyltransferase.
(TIF)

S4 Fig Glucose metabolism increases ROS/AMPK/EP300 activity in gastrointestinal cancer cells, whereas in liver cancer GYS2 expression prevents ROS accumulation in response to glucose 25 mM and associates with higher patient survival. **Related to Fig 4.** Cells starved of glucose for 24 h prior to re-feeding for the indicated times with 25 mM glucose or with indicated treatments were analyzed by western blotting in (A–B), (E), (H); by immunofluorescence in (D) and (G); or by flow cytometry in (F). (A) Effect of osmotic stress on AMPK/EP300 using 5 mM or 25 mM mannitol. (B) Inhibition of glucose metabolism with 5 mM 2-DG for 24 h, effect on AMPK/EP300. (C) Kaplan Meier analysis of the TCGA liver cancer patient cohort, ranked by GYS2 expression; GYS2 used as readout of glycogen synthesis capacity. Survival of patients with high and low GYS2 expression, red and blue lines, respectively. *P =* 0.0003872. (D) Accumulation of ROS in response to glucose or H_2_O_2_ as positive control, analyzed by DCF-DA (0.5 μM) labeling followed by immunofluorescence of indicated cell lines. H_2_O_2_ (100 μM) was added for the last 30 min as positive control of ROS signaling. (E) Time course to compare pAMPK (T172) induction by glucose in gastrointestinal cancer cells but not in liver cancer cells. Positive control of increased ROS, by exposure to H_2_O_2_ (100 μM) for the last 30 min, induce pAMPK (T172) in HCT 116 and Hep G2; pERK 1/2: positive control. Representative western blots and statistical analysis. (F) GYS2 depletion in liver cancer cells allows ROS accumulation in response to glucose 25 mM. Cells transfected with control or GYS2-specific siRNA for 48 h were starved of glucose 24 h. ROSs were accumulated in GYS2-depleted HepG2 liver cancer cells upon culture with 25 mM glucose for another 24 h measured by flow cytometry as in Fig 4E. (G) Immunofluorescence as in (D); where indicated, cells were pre-treated with CoQ10 (10 μM) for 12 h before glucose starvation. ROSs shown as green label. (H) CoQ10 interferes with EP300-driven H3K9 acetylation by glucose/ROS/AMPK. Pre-treatment with CoQ10 (10 μM) was for 12 h. Statistical analysis was performed in all cases after quantification of *n* ≥ 3 independent experiments by one-way ANOVA. Values represent mean ± SEM. *P < 0.05; **P < 0.01; ***P < 0.001. S1 Data presents individual data and find underlying raw images at S1 Raw Images. 2-DG, 2-Deoxy-D-glucose; ACC1, Acetyl-Coenzyme A Carboxilase 1; AMPK, AMP-activated protein kinase; Cnt, Control; CoQ, Coenzyme Q10; DCF-DA, 2’7’-dichlorodihydrofluorescein diacetate; EP300, Histone acetyltransferase p300; ERK, extracellular signal-regulated kinase 1; GAPDH, Glyceraldehyde 3-phosphate dehydrogenase; GYS2, Glycogen Synthase 2; H3K9 Ace, Histone H3 Lysine 9 acetylated
(TIF)

S5 Fig Glucose accelerates cell cycle and increases proliferation in gastrointestinal cancer cells. **Related to Fig 5.** Cells were cultured as indicated previously. (A) Flow cytometry analysis of cell cycle effects of glucose. Numbers correspond to the percentage of cells in the indicated phases expressed as mean ± SEM. Statistical analysis of 3 independent experiments; *P < 0.05; **P < 0.01; ***P < 0.001 by Student *t* test. (B) Proliferation of STC-1, HCT 116, or Caco-2 gastrointestinal cancer cells in the absence or presence of indicated glucose concentrations. Statistical analysis of *n* ≥ 3 independent experiments by one-way ANOVA (STC-1 and HCT 116) or Student *t* test (Caco-2; *P < 0.05; **P < 0.01; ***P < 0.001. Individual data can be found as S1 Data and underlying raw images at S1 Raw Images.
(TIF)

S1 Table Participant details. Clinicopathologic characteristics of colorectal cancer patients included in the study. DB, diabetes mellitus; N, number of patients; pT, tumor stage; pN, lymph node affection.
(DOCX)

S2 Table Materials. (DOCX)

S1 Data Data underlying Figs 1–7 and S1–S5 Figs.
(XLSX)

S1 Raw Images Original gel and images contained in this manuscript. (PDF)

## Abbreviations

8-OHdG: 8-hydroxy-2'-deoxyguanosine
2-DG: 2-deoxy-glucose
A76: AMPK inducer A-769662
Ace-Lys: acetyl-lysine
ACC1: Acetyl-Coenzyme A Carboxilase 1
AICAR: 5-aminoimidazole-4-carboxamide ribonucleotide
AKT: Serine-Threonine Kinase AKT or PKB
AMPK: AMP-activated protein kinase
AOM: azoxymethane
ATM: Serine-protein kinase ATM (Ataxia telangiectasia mutated)
Bro: Bromodomain
CBP: CREB-binding protein
CE: cytoplasmic extract
CGDS-R: R-Based package for accessing the Cancer Genomics Data Server
CHX: cycloheximide
CoQ10: Coenzyme Q10
CREB: Cyclic-AMP-Response Element Binding protein
Ct: Carboxy terminus
DCF-DA: 2’7’-dichlorodihydrofluorescein diacetate
DSS: Dextran Sodium Sulfate
EP300: Histone acetyltransferase p300
ERK: extracellular signal-regulated kinase 1
FBP: fructose-1,6-bisphosphate
FT: flow through
GAPDH: Glyceraldehyde 3-phosphate dehydrogenase
GSEA: Gene Set Enrichment Analysis
GSK3β: glycogen synthase kinase 3β
GYS2: Glycogen Synthase 2
H3K9: Histone H3 Lysine 9
IRB: Institutional Review Board
KAT: lysine acetyl transferase catalytic center
KIX: Nuclear receptor and CREB interacting domains
KO: knockout
MAPK: mitogen-activated protein kinase
MSigDB: Molecular Signatures Database
mTORC1: mammalian Target Of Rapamycin Complex 1
P38: Mitogen-activated protein kinase P38
PAS: Periodic Acid-Schiff
PAS-D: Periodic Acid-Schiff with alpha amylase
PCAF: P300/CBP-associated factor
PhD: PhD finger
PKC: protein kinase C
PPARγ: Peroxisome Proliferator-Activated Receptor gamma
PTM: post translational modification
qRT-PCR: quantitative Reverse Transcription Polymerase Chain Reaction
RAR: All-trans Retinoic Acid Receptor
ROS: reactive oxygen species
RSEM: RNA Sequencing by Expectation Maximization
RXR: 9-cis Retinoic Acid Receptor
SC: Scramble Control
siRNA: small interfering RNA
TBP: TATA-box-Binding Protein
TCGA: The Cancer Genome Atlas
TMA: tissue microarray
TR: Thyroid Hormone Receptor
VDR: Vitamin D Receptor
WT: wild type
