# Supplementary figures and images for: Paradoxical activation of AMPK by glucose drives selective EP300 activity in colorectal cancer
Source: PLoS Biol. 2020 Jun 30;18(6):e3000732. doi: 10.1371/journal.pbio.3000732 (PMC7326158; doi:10.1371/journal.pbio.3000732)

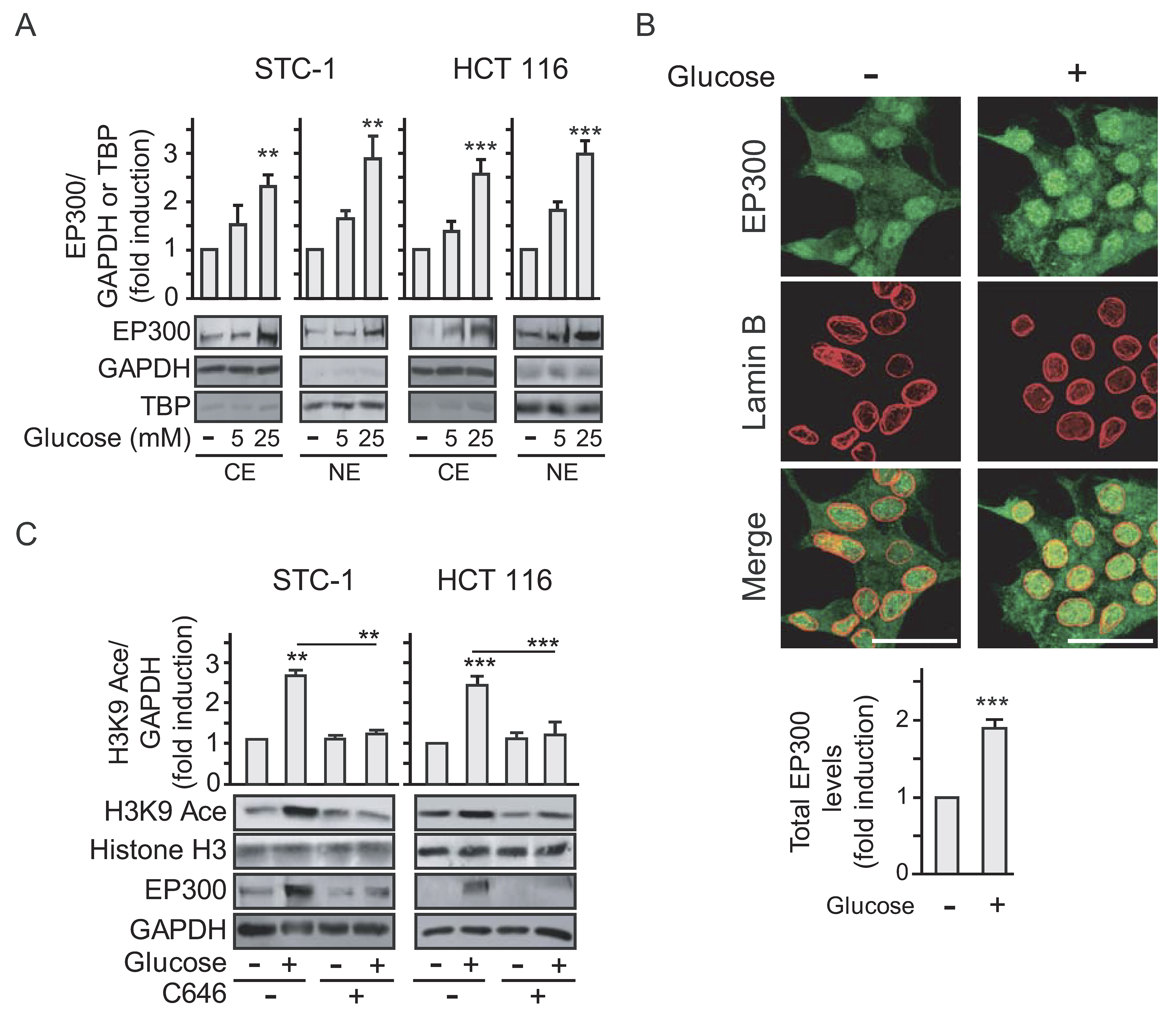

Supplement: S1 Fig — Related to Fig 1. Cells were starved of glucose for 36 h (−) before addition of 5 mM or 25 mM glucose for 24 h (A) or glucose 25 mM for 24 h (+) (B–D). C646 (5 μM) was added for 24 h where indicated. (A) Representative western blots of EP300 and statistical analysis of cytoplasmic (CE) or nuclear (NE) extracts from indicated cell lines. GAPDH or TBP are loading controls for cytoplasmic and nuclear fractions, respectively. (B) Confocal immunofluorescence images of STC-1 cells using indicated antibodies (scale bars represent 25 μm) and quantification of fluorescence intensity using ImageJ software (lower panel); for each experiment, 3 different fields were evaluated per slide. (C) Representative western blot and statistical analysis of the correlation between glucose induction of EP300 and H3K9 acetylation in gastrointestinal cancer cell lines. The selective EP300 inhibitor C646 abolishes EP300 and H3K9 acetylation. Statistical analysis by one-way ANOVA (A) and (C) or Student t test (B); n ≥ 3; *P < 0.05, **P < 0.01; ***P < 0.001. See individual data at S1 Data and underlying raw images at S1 Raw Images. CE, cytoplasmic extracts; CRC, colorectal cancer; EP300, Histone acetyltransferase p300; GAPDH, Glyceraldehyde 3-phosphate dehydrogenase; H3K9 Ace, Histone H3 Lysine 9 acetylated; NE, Nuclear extracts; TBP, TATA-box-Binding Protein. (TIF) [file pbio.3000732.s001.tif]

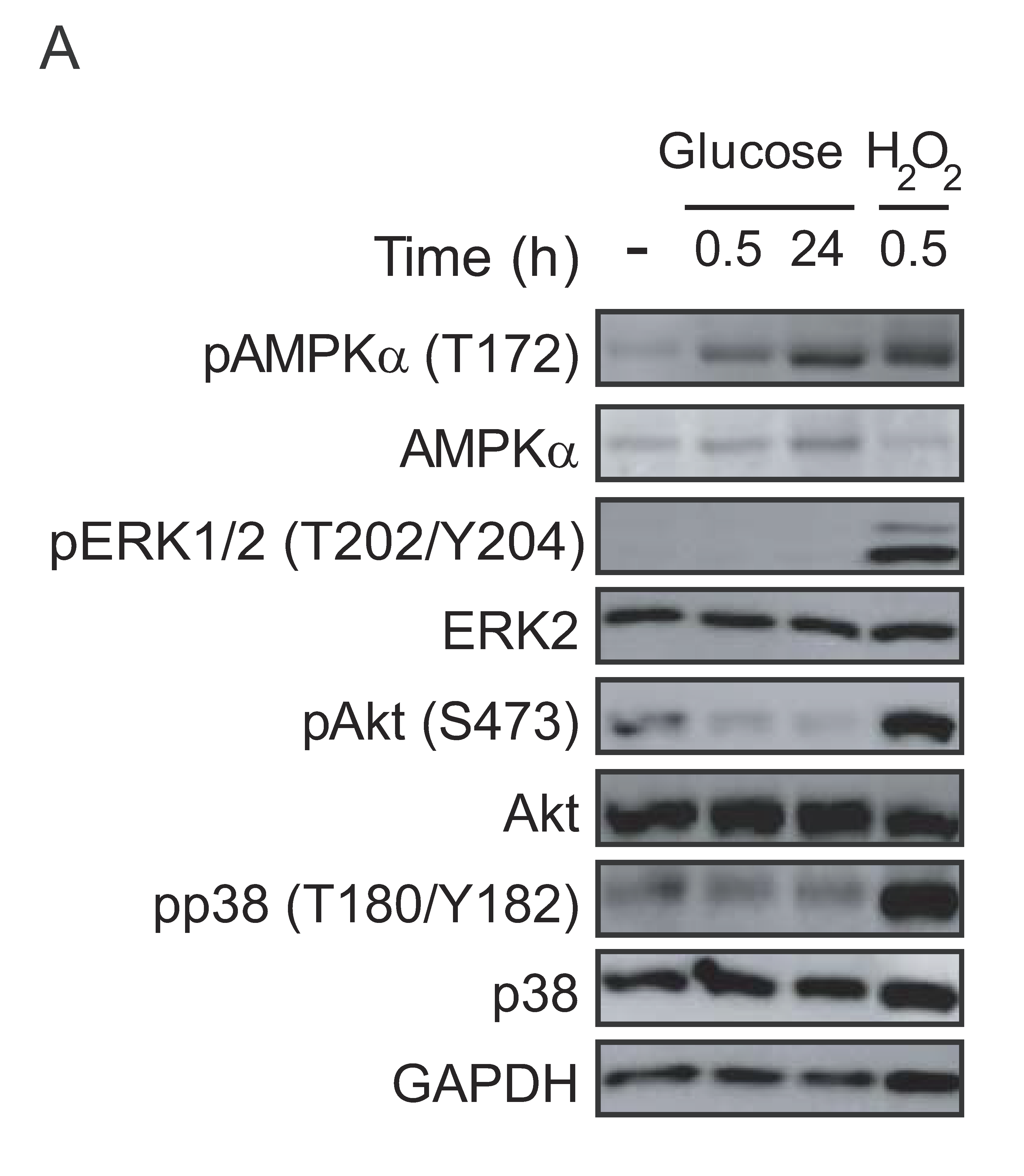

Supplement: S2 Fig — Related to Fig 2. (A) Kinase induction was analyzed in STC-1 whole cell extracts; H2O2 (100 μM), was used as positive control for induction of pERK, pAKT, pp38, and pAMPK activation. GAPDH, loading control. Kinases previously reported to modify EP300 were studied. AKT, Serine-Threonine Kinase AKT or PKB; AMPK, AMP-activated protein kinase; ERK, ERK, extracellular signal-regulated kinase 1; GAPDH, Glyceraldehyde 3-phosphate dehydrogenase; P38, Mitogen-activated protein kinase P38 (TIF) [file pbio.3000732.s002.tif]

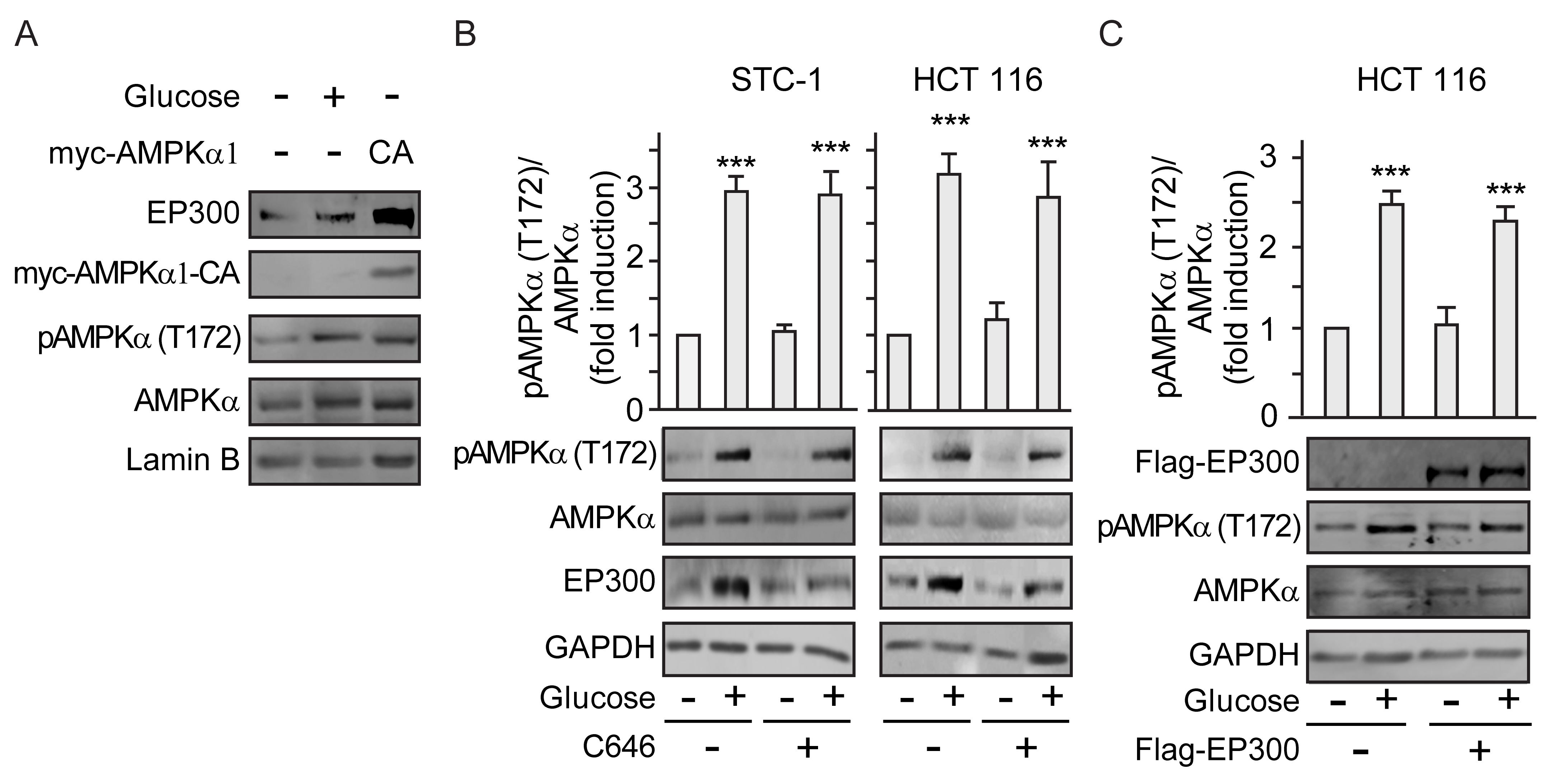

Supplement: S3 Fig — Related to Fig 3. (A) Whole cell extracts of STC-1 cells transfected with a Myc-tagged deletion mutant of AMPK catalytic subunit that is constitutively active (CA) for 48 h and then starved of, or treated with, glucose (25 mM) for 24 h. Note the molecular weight of the myc-AMPKα1-CA is 37 KDa versus 63 KDa of the full length since it contains only amino acids 1–312 [32]. (B) The EP300 inhibitor C646 (5 μM) was added to STC-1 or HCT 116 cells cultured as previously described for the last 24 h. C646 inhibition did not abolish AMPK induction by glucose. (C) HCT 116 cells transfected with control or pCDNA3-Flag-EP300 expression vector were cultured as previously described to analyze whether EP300 alters glucose induction of AMPK. Statistical analysis (B–C) by one-way ANOVA; n ≥ 3; *P < 0.05, **P < 0.01; ***P < 0.001. Individual data can be found as S1 Data and underlying raw images at S1 Raw Images. AMPK, AMP-activated protein kinase; GAPDH, Glyceraldehyde 3-phosphate dehydrogenase; EP300, Histone acetyltransferase. (TIF) [file pbio.3000732.s003.tif]

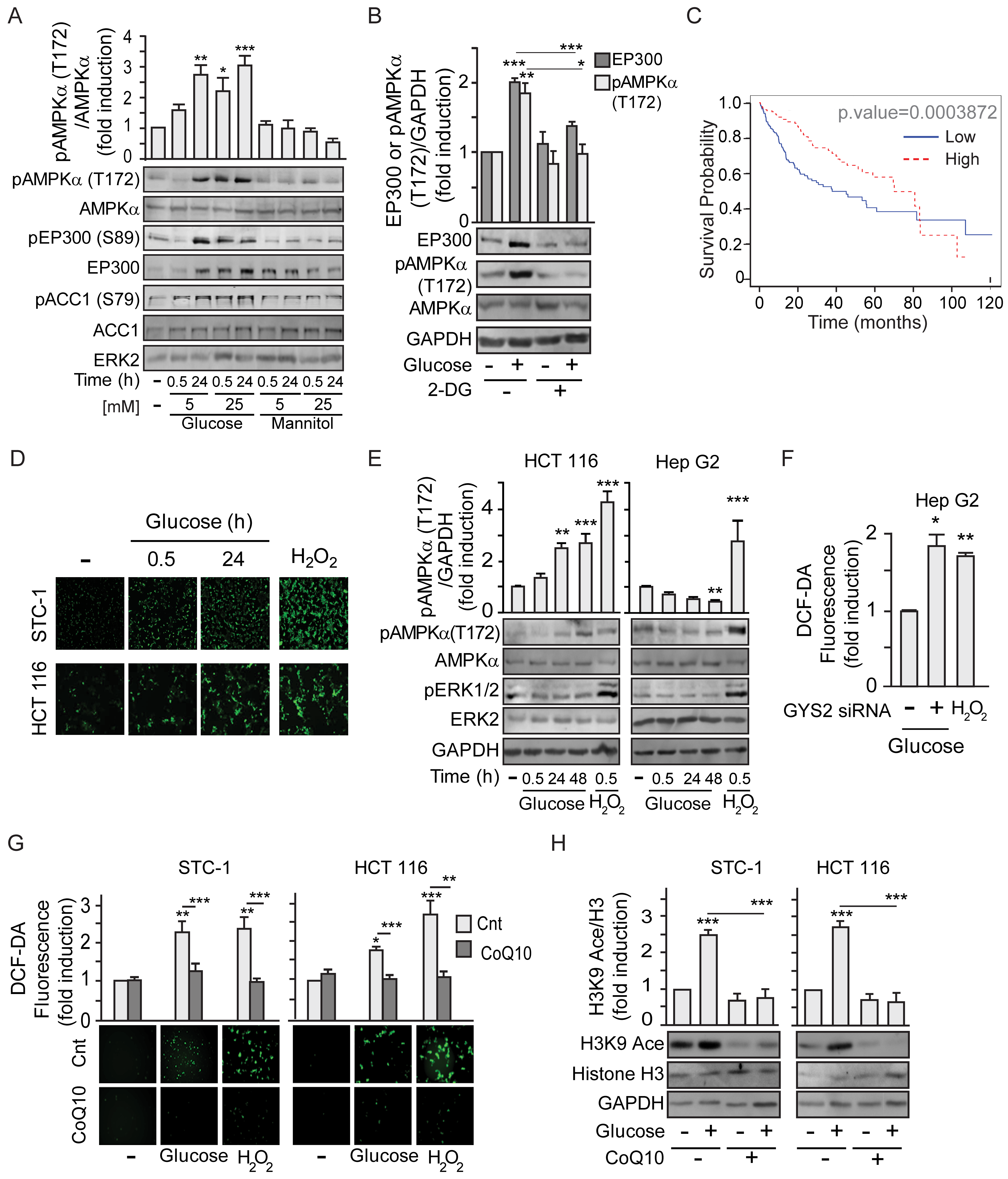

Supplement: S4 Fig — Related to Fig 4. Cells starved of glucose for 24 h prior to re-feeding for the indicated times with 25 mM glucose or with indicated treatments were analyzed by western blotting in (A–B), (E), (H); by immunofluorescence in (D) and (G); or by flow cytometry in (F). (A) Effect of osmotic stress on AMPK/EP300 using 5 mM or 25 mM mannitol. (B) Inhibition of glucose metabolism with 5 mM 2-DG for 24 h, effect on AMPK/EP300. (C) Kaplan Meier analysis of the TCGA liver cancer patient cohort, ranked by GYS2 expression; GYS2 used as readout of glycogen synthesis capacity. Survival of patients with high and low GYS2 expression, red and blue lines, respectively. P = 0.0003872. (D) Accumulation of ROS in response to glucose or H2O2 as positive control, analyzed by DCF-DA (0.5 μM) labeling followed by immunofluorescence of indicated cell lines. H2O2 (100 μM) was added for the last 30 min as positive control of ROS signaling. (E) Time course to compare pAMPK (T172) induction by glucose in gastrointestinal cancer cells but not in liver cancer cells. Positive control of increased ROS, by exposure to H2O2 (100 μM) for the last 30 min, induce pAMPK (T172) in HCT 116 and Hep G2; pERK 1/2: positive control. Representative western blots and statistical analysis. (F) GYS2 depletion in liver cancer cells allows ROS accumulation in response to glucose 25 mM. Cells transfected with control or GYS2-specific siRNA for 48 h were starved of glucose 24 h. ROSs were accumulated in GYS2-depleted HepG2 liver cancer cells upon culture with 25 mM glucose for another 24 h measured by flow cytometry as in Fig 4E. (G) Immunofluorescence as in (D); where indicated, cells were pre-treated with CoQ10 (10 μM) for 12 h before glucose starvation. ROSs shown as green label. (H) CoQ10 interferes with EP300-driven H3K9 acetylation by glucose/ROS/AMPK. Pre-treatment with CoQ10 (10 μM) was for 12 h. Statistical analysis was performed in all cases after quantification of n ≥ 3 independent experiments by one-way ANOV [file pbio.3000732.s004.tif]

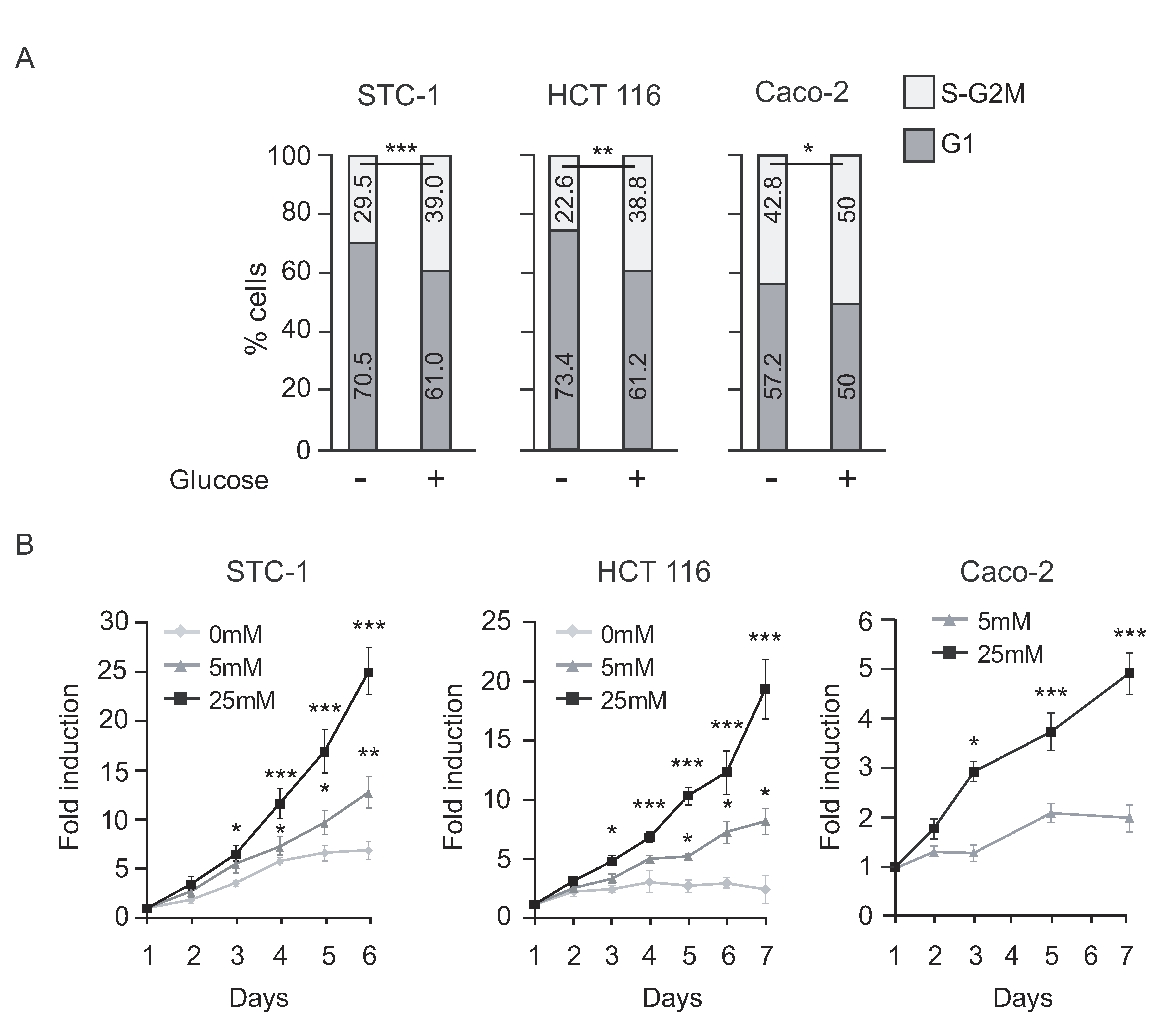

Supplement: S5 Fig — Related to Fig 5. Cells were cultured as indicated previously. (A) Flow cytometry analysis of cell cycle effects of glucose. Numbers correspond to the percentage of cells in the indicated phases expressed as mean ± SEM. Statistical analysis of 3 independent experiments; *P < 0.05; **P < 0.01; ***P < 0.001 by Student t test. (B) Proliferation of STC-1, HCT 116, or Caco-2 gastrointestinal cancer cells in the absence or presence of indicated glucose concentrations. Statistical analysis of n ≥ 3 independent experiments by one-way ANOVA (STC-1 and HCT 116) or Student t test (Caco-2; *P < 0.05; **P < 0.01; ***P < 0.001. Individual data can be found as S1 Data and underlying raw images at S1 Raw Images. (TIF) [file pbio.3000732.s005.tif]
