# Supplementary material for: Paradoxical activation of AMPK by glucose drives selective EP300 activity in colorectal cancer
Source: PLoS Biol. 2020 Jun 30;18(6):e3000732. doi: 10.1371/journal.pbio.3000732 (PMC7326158; doi:10.1371/journal.pbio.3000732)
Supplement: S1 Table — Clinicopathologic characteristics of colorectal cancer patients included in the study. DB, diabetes mellitus; N, number of patients; pT, tumor stage; pN, lymph node affection. (DOCX) [file pbio.3000732.s006.docx]

**S1_Table. Subject details**

Clinico pathologic characteristics of colorectal cancer patients included in the study; N: number of patients; pT: Tumor stage; pN:lymph node affection; DM: Diabetes Mellitus.

| **Patients characteristics** | | **N (%)** |  | **Patients characteristics** | | **N (%)** |
| --- | --- | --- | --- | --- | --- | --- |
| **Median Age-years (range )** | | 74 (94-51) |  | **pT** |  |  |
| **Gender** / Male | | 57 (60%) |  | T1 |  | 3 (3%) |
| **Gender** / Female | | 38 (40%) |  | T2 |  | 30 (32%) |
| **Mucinous** Yes | | 9 (10%) |  | T3 |  | 61 (64%) |
| **Mucinous** No | | 86 (90%) |  | T4 |  | 1 (1%) |
| **Tumour Location** | |  |  | **pN** / N0 |  | 95 (100%) |
| Caecum |  | 13 (14%) |  | **DM Type II** |  |  |
| Right |  | 25 (26%) |  | Yes |  | 8 (9%) |
| Transverse |  | 7 (8%) |  | No |  | 42 (44%) |
| Left |  | 5 (5%) |  | N/A |  | 45 (47%) |
| Sigma |  | 24 (25%) |  | **Treatment DM** | |  |
| Rectum |  | 21 (21%) |  | Metformin |  | 6 (75%) |
| **Grade** |  |  |  | Insulin |  | 1 (12,5%) |
| Well differentiated | | 18 (19%) |  | Gliclazide |  | 1 (12,5%) |
| Moderately differentiated | | 69 (73%) |  |  |  |  |
| Poorly differentiated | | 8 (8%) |  |  |  |  |
