# Supplementary material for: Paradoxical activation of AMPK by glucose drives selective EP300 activity in colorectal cancer
Source: PLoS Biol. 2020 Jun 30;18(6):e3000732. doi: 10.1371/journal.pbio.3000732 (PMC7326158; doi:10.1371/journal.pbio.3000732)
Supplement: S2 Table — (DOCX) [file pbio.3000732.s007.docx]

**S2_Table: Materials.**

| **Antibodies** | **SOURCE** | **Catalog number** |
| --- | --- | --- |
| Rabbit Polyclonal Acetyl lysine | Cell Signaling | Cat # 9441 |
| Rabbit Polyclonal pAMPK (T172) | Cell Signaling | Cat # 2531 |
| Rabbit Polyclonal AMPKα | Cell Signaling | Cat # 2532 |
| Rabbit monoclonal Histone H3K9-Ace (C5B11) | Cell Signaling | Cat # 9649 |
| Rabbit Polyclonal Histone H3 | Cell Signaling | Cat # 9715S |
| Rabbit Polyclonal pACC1 (S79) | Cell Signaling | Cat # 3661 |
| Rabbit Polyclonal ACC1 | Cell Signaling | Cat # 3662 |
| Rabbit monoclonal pAKT (S473) | Cell Signaling | Cat # 4058 |
| Rabbit Polyclonal pp38 (T180/Y182) | Cell Signaling | Cat #9211S |
| Rabbit Polyclonal p38 | Cell Signaling | Cat #9212S |
| Goat Polyclonal AKT (C-20) | Santa Cruz Bio | SC-1618 |
| Rabbit Polyclonal p-p300 (S89) | Santa Cruz Bio | SC-130210 |
| Rabbit Polyclonal p300 (N-15) | Santa Cruz Bio | SC-584 |
| Rabbit Polyclonal p300 (C20) | Santa Cruz Bio | SC-585 |
| Rabbit Polyclonal CBP (C20) | Santa Cruz Bio | SC-583 |
| Rabbit Polyclonal TFIID-TBP (N12) | Santa Cruz Bio | SC-204 |
| Rabbit Polyclonal ERK (C14) | Santa Cruz Bio | SC-154 |
| Rabbit Polyclonal β-CATENIN (C18) | Santa Cruz Bio | SC-1496-R |
| Rabbit Polyclonal GYS2 | ThermoFisher | PA5-42763 |
| Mouse Monoclonal β-Actin | Santa Cruz Bio | SC-47778 |
| Goat polyclonal 8 Hydroxyguanosine (8-OHdG) | Abcam | ab10802 |
| Rabbit Polyclonal pp300 (S89) | Invitrogen | PA512652 |
| Rabbit monoclonal pAMPK (T172) (D4D6D) | Cell Signaling | Cat # 50081 |
| Goat Polyclonal LAMIN B (C20) | Santa Cruz Bio | SC-6216 |
| Mouse Monoclonal MYC (9E10) | Santa Cruz Bio | SC-40 |
| Mouse Monoclonal GAPDH | Sigma-Aldrich | # G8795 |
| Mouse Monoclonal M2 FLAG | Sigma-Aldrich | # F 3165 |
| Rabbit Monoclonal pERK1/2 (T202/Y204) | Millipore | Cat. # 05-797R |
| Goat Anti-Rabbit IgG (H+L) HRPO | BIO-RAD | Cat #170-6515 |
| Goat Anti-Mouse IgG (H+L) HRPO | BIO-RAD | Cat #170-6516 |
| Rabbit-Anti Goat IgG (H+L) HRPO | BIO-RAD | Cat #172-1034 |
| Donkey Anti-Rabbit- AlexaFluor488 | Invitrogen | A21206 |
| Donkey Anti-Goat-AlexaFluor647 | Invitrogen | A21447 |
| **Oligonucleotides** | | |
| 5´ TCCCCAGATGGGAGGACAAA-3’ | This paper | p300 RT/PCR-Forwards |
| 5’- TCCACCAGGAACTGCCTTTT-3’ | This paper | p300 RT/PCR Reverse |
| 5’-AAGATCGTCGCCACCTGG-3’ | This paper | Cyclin D1 RT/PCR forwards |
| 5’-GGAAGACCTCCTCCTCGCAC-3’ | This paper | Cyclin D1 RT/PCR Reverse |
| 5’-CTTCTCTCCGTCCTCGGATTCT-3’ | This paper | Myc RT/PCR forwards |
| 5’-GAAGGTGATCCAGACTCTGACCTT-3’ | This paper | Myc RT/PCR Reverse |
| 5′ AGTCCCTGCCCTTTGTACACA 3′ | This paper | 18s RT/PCR Forwards |
| 5′ GCCTCACTAAACCATCCAATCG 3′ | This paper | 18s RT/PCR Reverse |
| siRNA Mouse siAMPKα1/2 | Santa Cruz Bio | SC-45313 |
| siRNA Human siAMPKα1/2 | Santa Cruz Bio | SC-45312 |
| ON-TARGETplus human GYS2 siRNA | Dharmacon | Cat# 2998 |
| r(UCCAUUCUGGUGCCACCAC)d(TT) and r(GUGGUGGCACCAGAAUGGA)d(TT) | Qiagen | siRNA Human β-catenin |
| AllStars Negative Control siRNA | Qiagen 1027420 | Cat# SI03650318 |
| **Recombinant DNA** | | |
| Flag-p300 | Donated by Dr. A. Schepsky | N/A |
| Myc-AMPKα1 CA | Donated by Dr. D. Carling | N/A |
| Bacterial and Virus Strains | | |
| E. coli 5α | E. coli 5α | E. coli 5α |
| Chemicals, Peptides, and Recombinant Proteins | | |
| Glucose | Sigma-Aldrich | G8769 |
| 2-Deoxy-D-glucose | Sigma-Aldrich | D8375 |
| AICAR | Sigma-Aldrich | A9978 |
| Metformin | Sigma-Aldrich | PHR1084 |
| Compound C | Sigma-Aldrich | P5499 |
| H_2_O_2_ | Sigma-Aldrich | H1009 |
| Coenzyme Q10 | Sigma-Aldrich | C9538 |
| Mannitol | Sigma-Aldrich | M4125 |
| Cycloheximide | Sigma-Aldrich | Cat # 01810 |
| A-769662 | TOCRIS Biosc. | Cat # 3336/10 |
| Bradford | Sigma Aldrich | B6916 |
| BSA | Sigma Aldrich | A7906 |
| C646 | Calbiochem | Cat# 382113 |
| TRIzol reagent | Invitrogen | Cat#15596026 |
| Protease inhibitor Cocktail | Roche | Cat#04693132001 |
| 7-AAD | Santa Cruz Biotech | SC-221210 |
| JetPei PolyPlus reagent | Genycell Biotech | Cat# 101-10N |
| JetPrime PolyPlus reagent | Genycell Biotech | Cat # 114-01 |
| QuikChange Kit | Stratagene | Cat #200523 |
| DCF-DA | Invitrogen/ Molecular Probes | Ref C6827 |
| DYNAbeads Protein A | Invitrogen | Ref 10002D |
| DYNAbeads Protein G | Invitrogen | Ref 10004D |
| AOM | Sigma-Aldrich | A5486 |
| DSS | MP Biomedicals | Ref 160110 |
| Experimental Models: Cell Lines | | |
| STC-1 (Strain C57B1/6J) Mouse enteroendocrine tumor | Donated by Dr. Hanahann | RRID:MGI:4361452 |
| HCT 116 (Male) colorectal carcinoma | ATCC | Cat# CCL-247, RRID:CVCL_0291 |
| HT-29 (Female). Rectosigmoid adenocarcinoma | ATCC | Cat# HTB-38, RRID:CVCL_0320 |
| Caco-2 WT (Male) Colorectal adenocarcinoma | ATCC | Cat # HTB-37^™^  RRID; CVCL_0025 |
| Caco-2 SC | Olivier et al, 2019; Grenier et al- 2018 |  |
| Caco-2 AMPKα1/α2 KO | Olivier et al, 2019; Grenier et al- 2018 |  |
| LS 174T (Female) Dukes' type B,colorectal adenocarcinoma | Donated by Dr. A. Muñoz | RRID:CVCL_1384 |
| HIEC_6 (Female) Human intestinal epithelial |  | RRID:CVCL_6C21 |
| Hep G2 (Male) Hepatoblastoma | Donated by Dr. A, Sanchez-Pacheco | RRID: CVCL_0027 |
| AspC-1 (Female) pancreas adenocarcinoma. From metastasis. | ATCC | Cat# CRL-1682, RRID:CVCL_0152 |
| IGR 37 (Male). Melanoma. From metastasis. | DSMZ | Cat# ACC-237, RRID:CVCL_2075 |
| Raji. (Male). B-Cell Lymphoma | Donated by Dr J M Zapata | RRID:CVCL_2699 |

| Software and Algorithms | | |
| --- | --- | --- |
| LAS AF software | Leica | SP5 |
| 3730xl Analyzer | Applied Biosystem | ABI 3730XL |
| 7500 FAST v2.3 | Life Technologies | 7500 FAST |
| GraphPad Prism software | GraphPad Software | <https://www.graphpad.com> |
| Typhoon scanner control 3.0 | Applied Biosystem | Typhoon 9210 |
| ImageLab | Bio-Rad | ChemiDoc XRS+ System |
| CXP software | Becton-Dickinson | FACSCalibur |
| ImageJ software | <https://imagej.nih.gov/ij/download.html> | N/A |
| RNAseq data | cBioportal | <http://www.cbioportal.org> |
| Molecular Signatures | MSigDB | <http://www.broadinstitute.org/msigdb> |
