# Supplementary material for: Paradoxical activation of AMPK by glucose drives selective EP300 activity in colorectal cancer
Source: PLoS Biol. 2020 Jun 30;18(6):e3000732. doi: 10.1371/journal.pbio.3000732 (PMC7326158; doi:10.1371/journal.pbio.3000732)

Raw images of western blotting

Related to Fig 1A

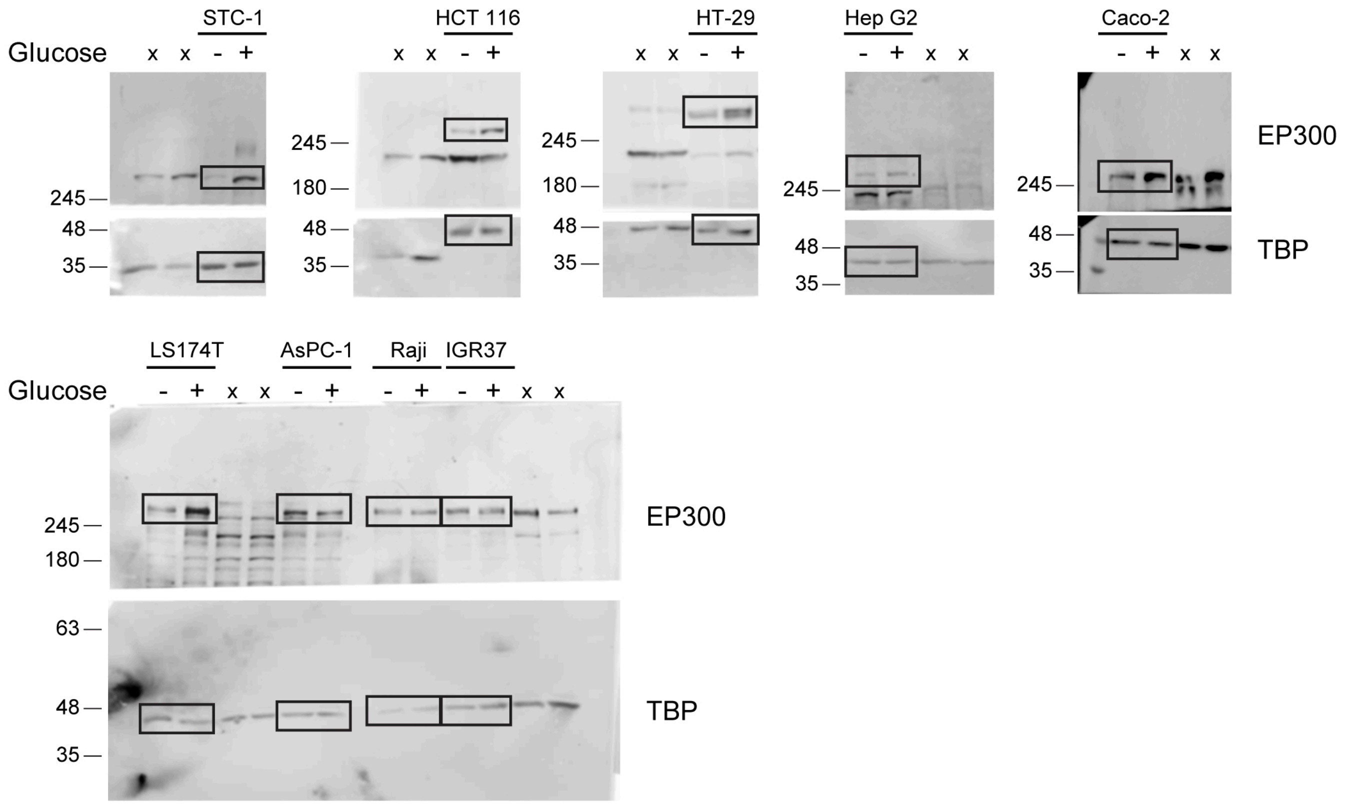

Related to Fig 1B

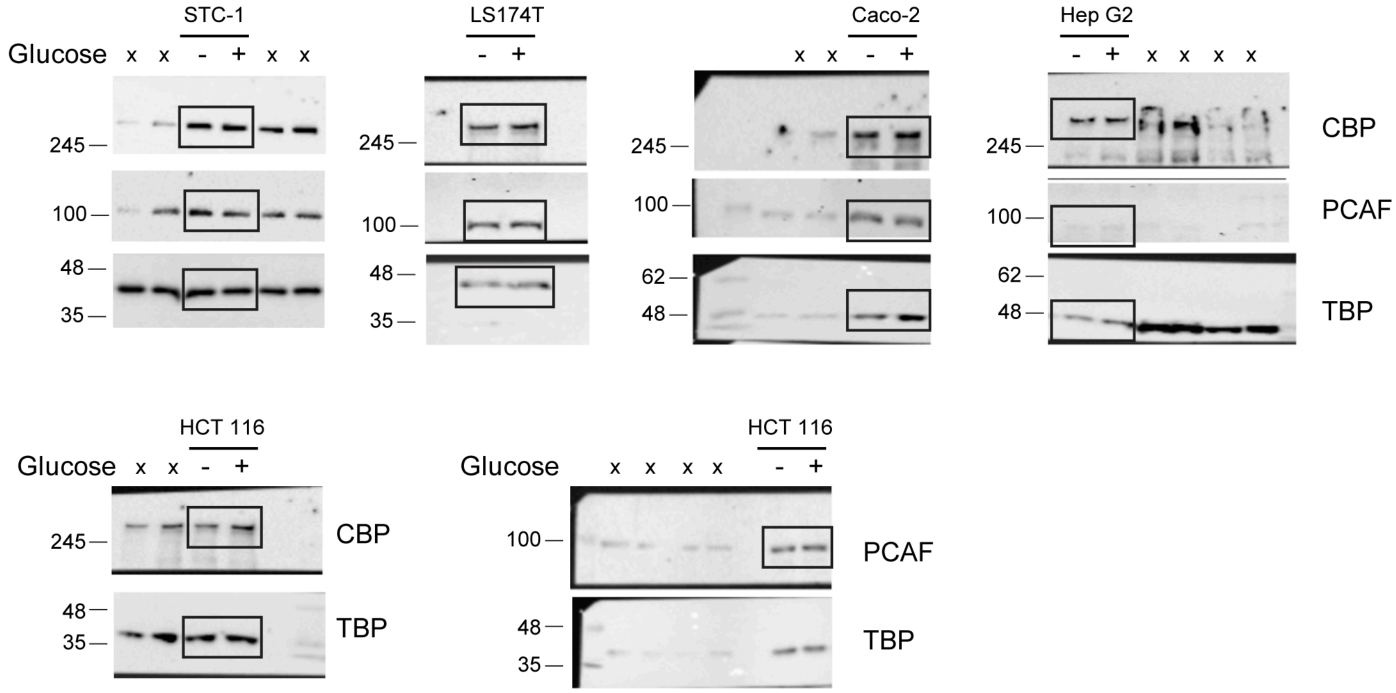

Related to Fig 1D

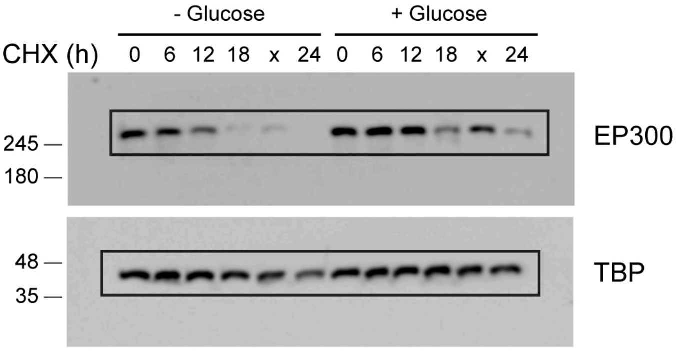

Related to Fig 1E

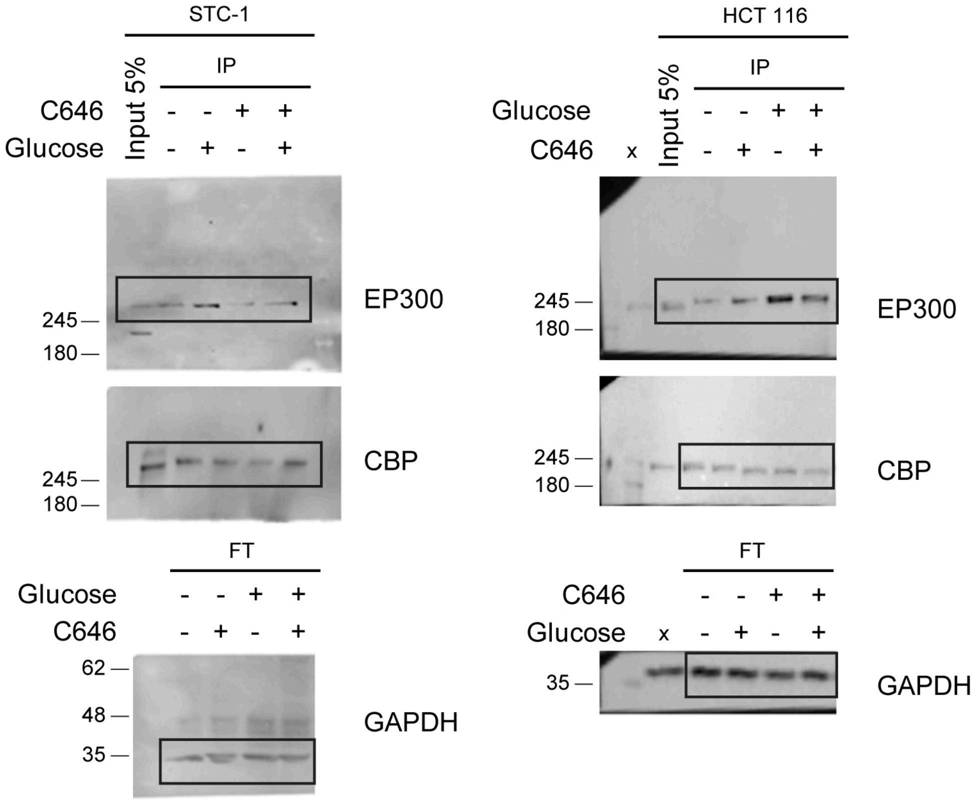

Related to Fig 2B

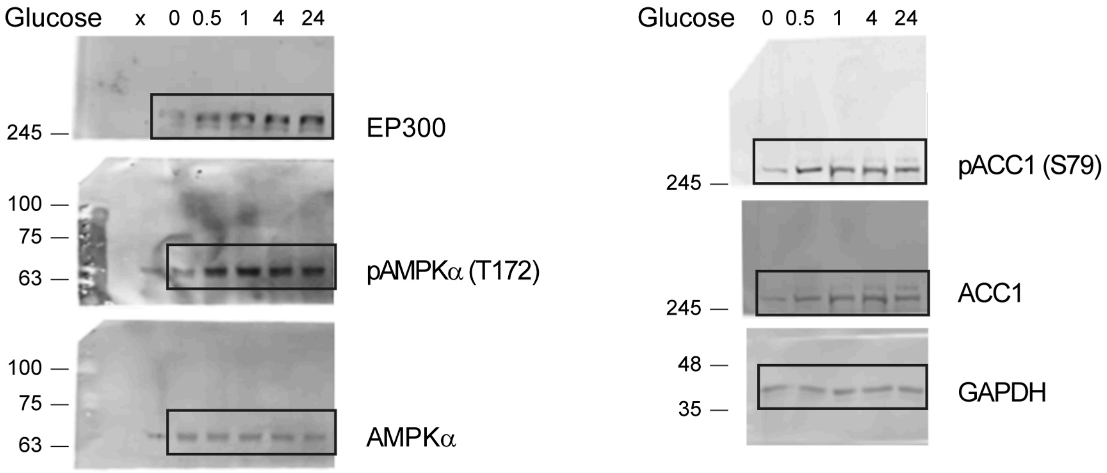

Related to Fig 2C

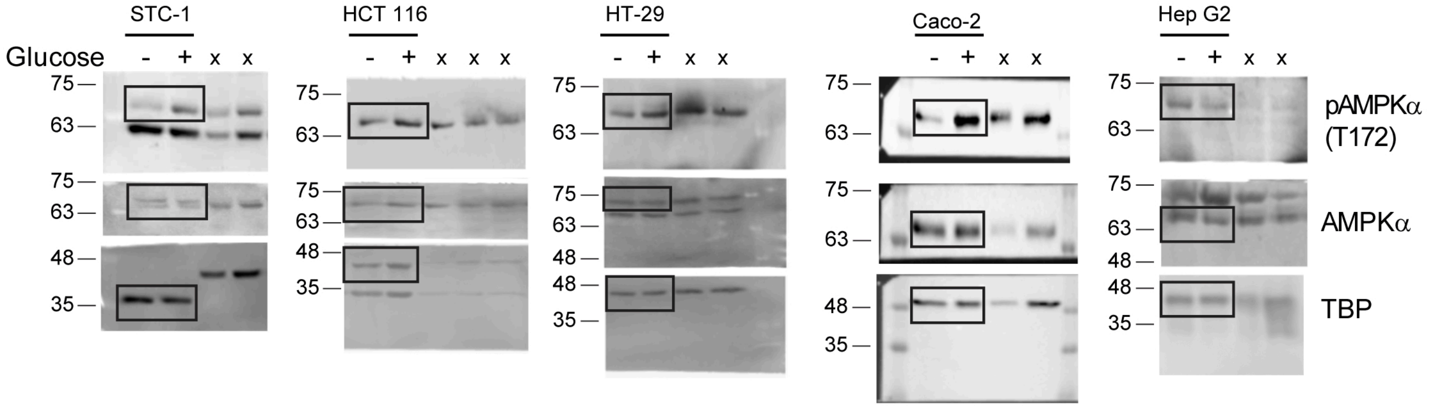

Related to Fig 2D

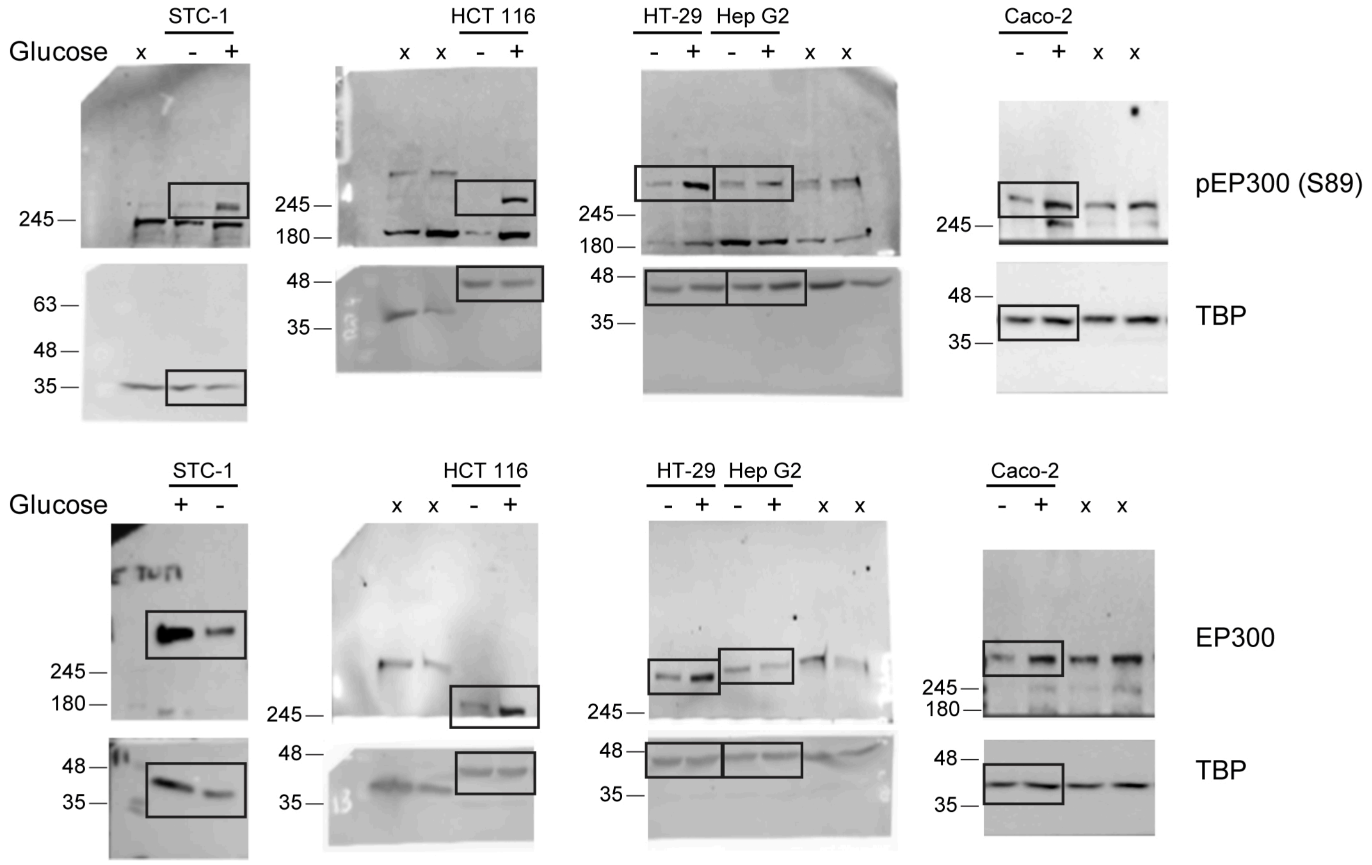

Related to Fig 2E

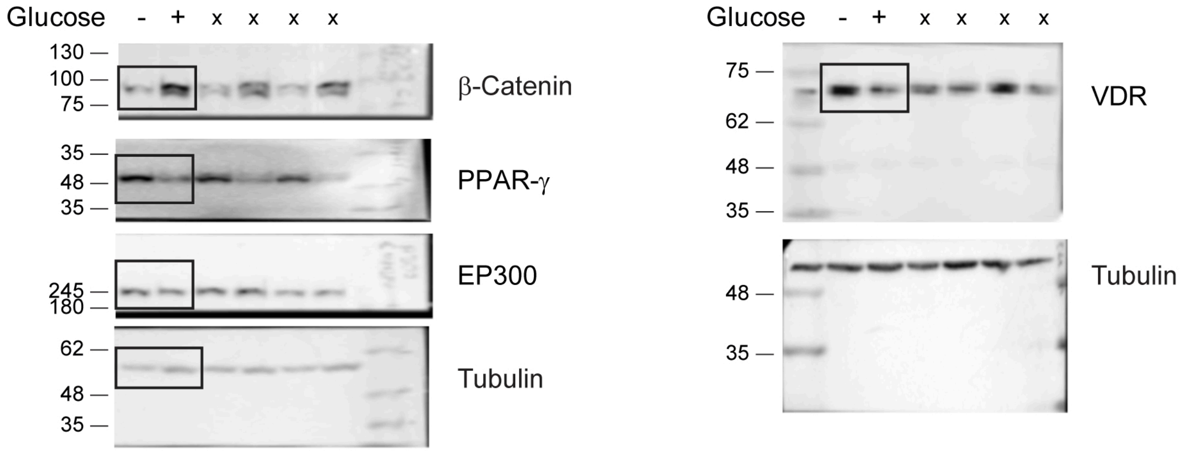

Related to Fig 3A

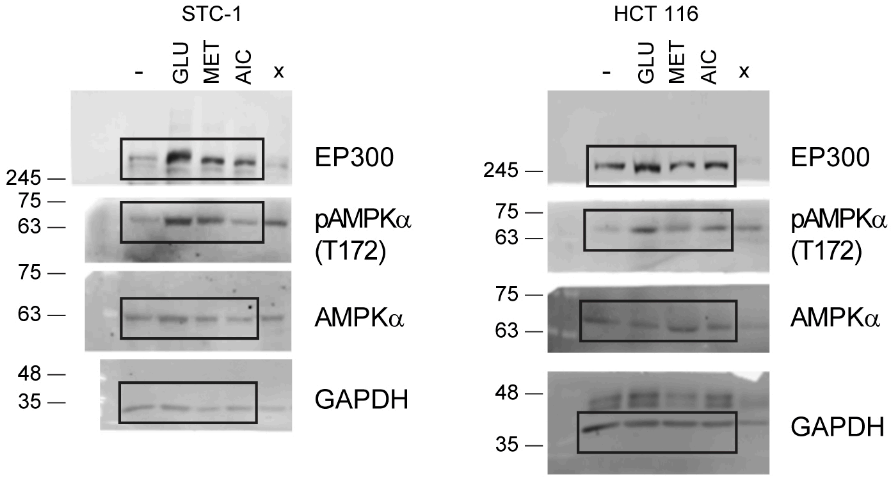

Related to Fig 3B

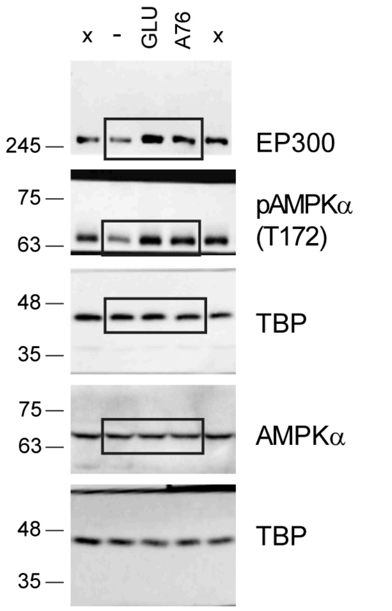

Related to Fig 3C

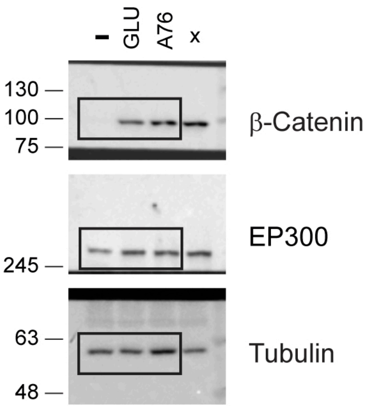

Related to Fig 3D

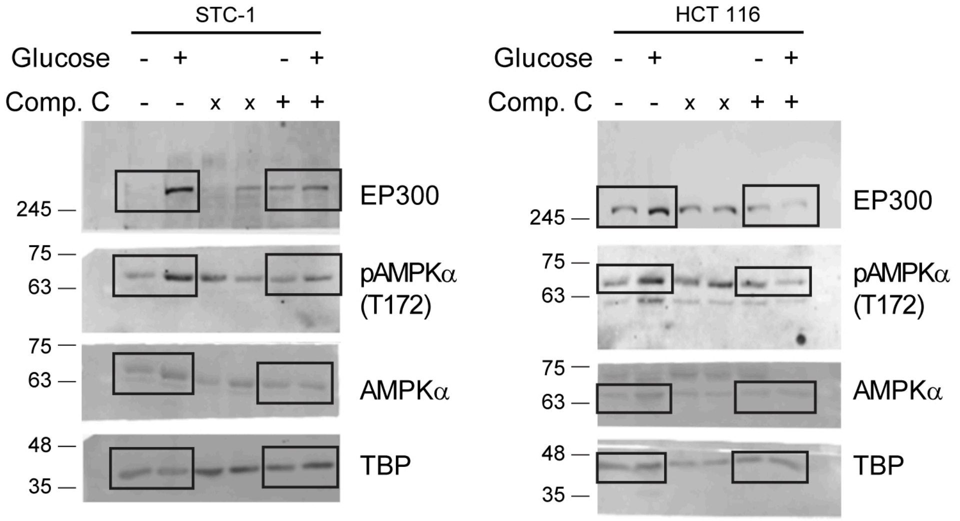

Related to Figure 3E

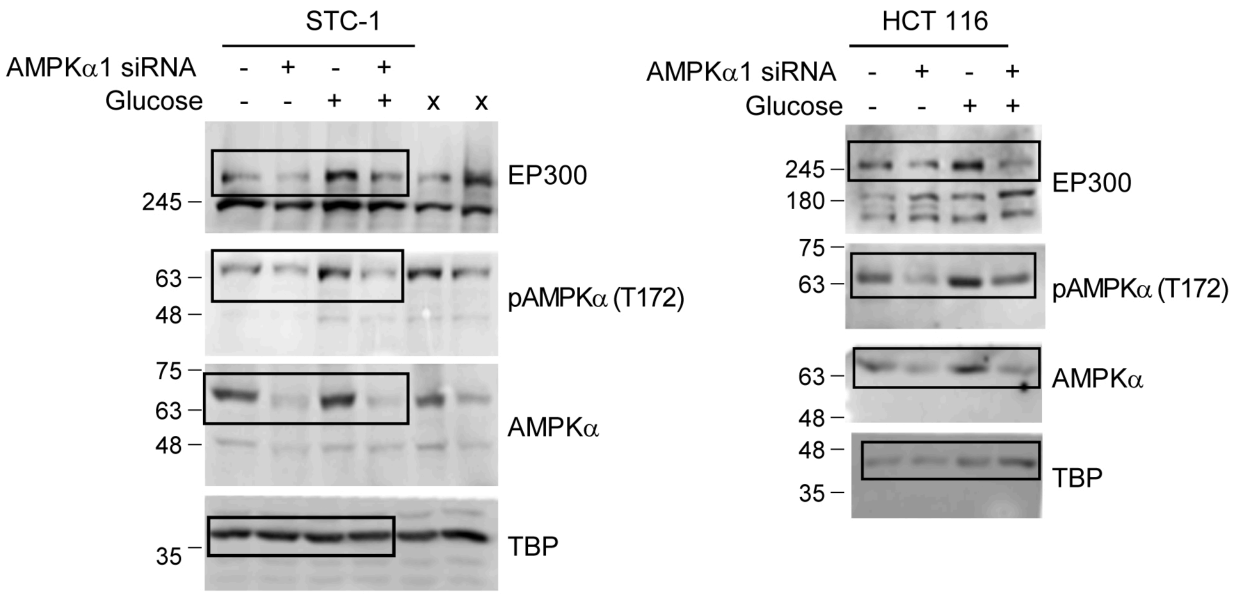

Related to Figure 3F

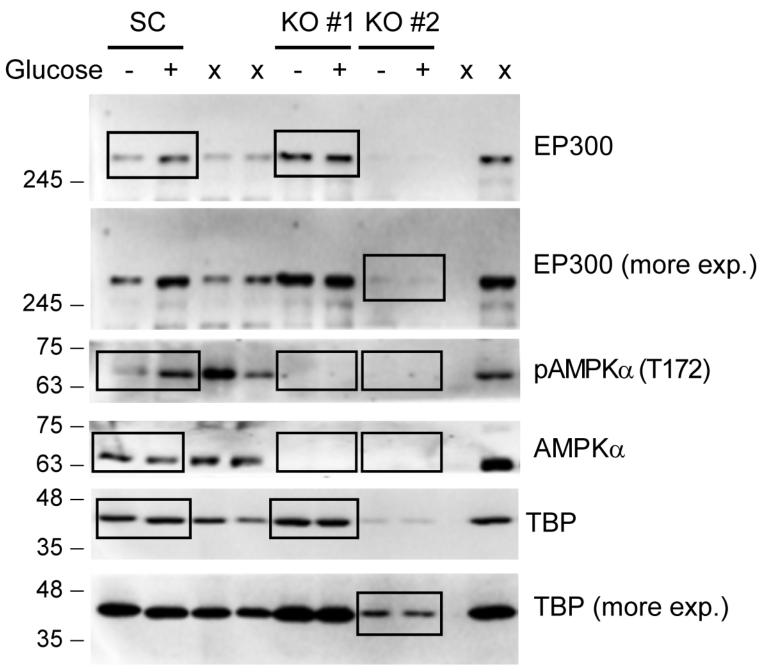

Related to Figure 4B

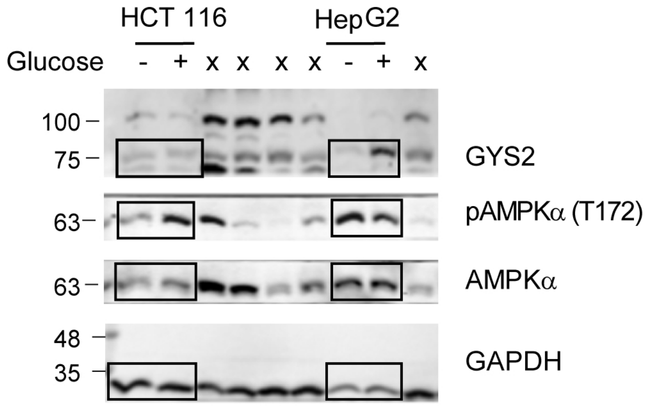

Related to Figure 4C

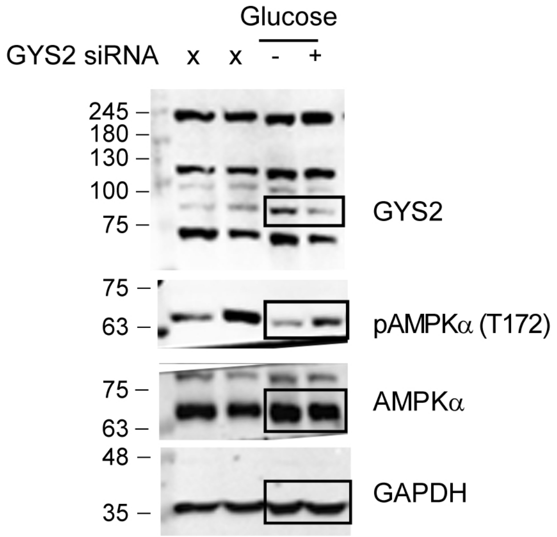

Related to Figure 4F

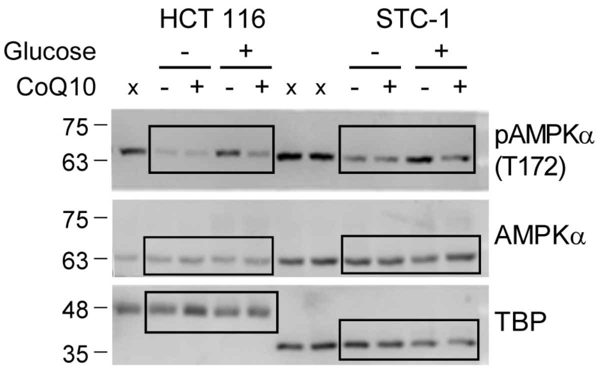

Related to Figure 4G

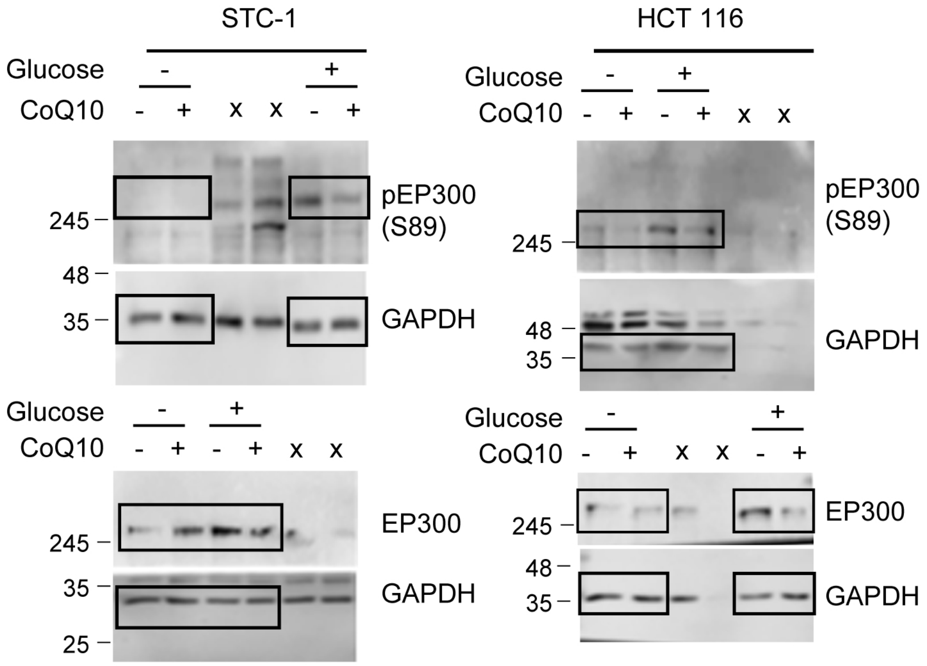

Related to Figure 5H

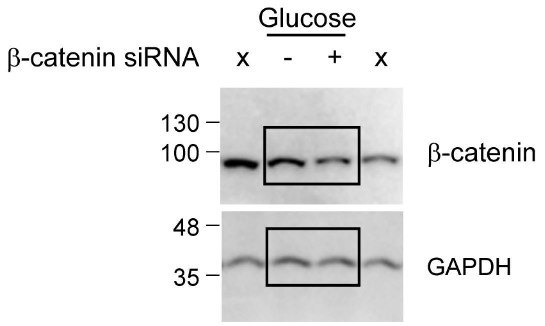

Related to Figure S1A

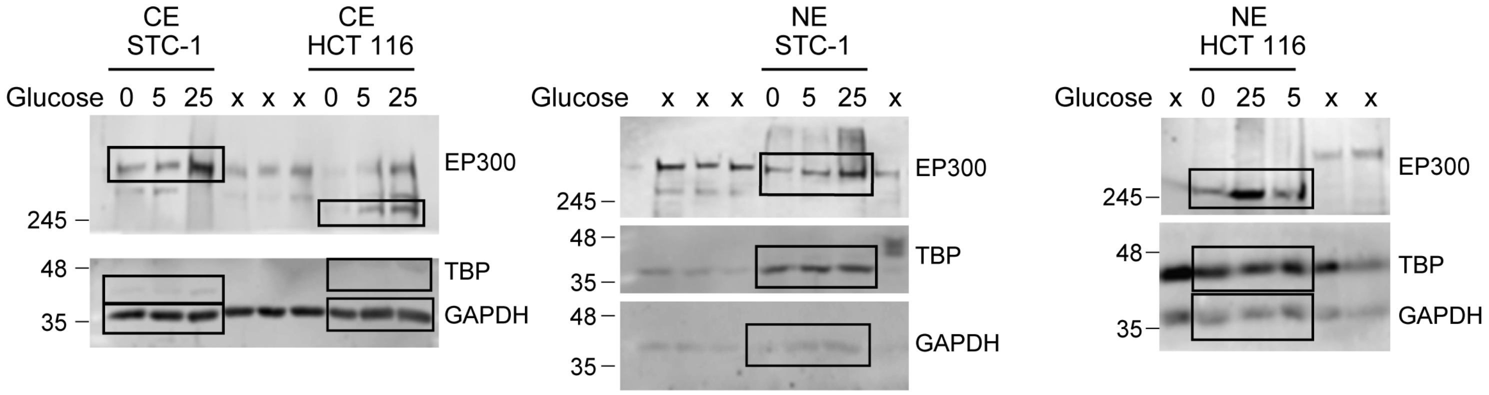

Related to Figure S1C

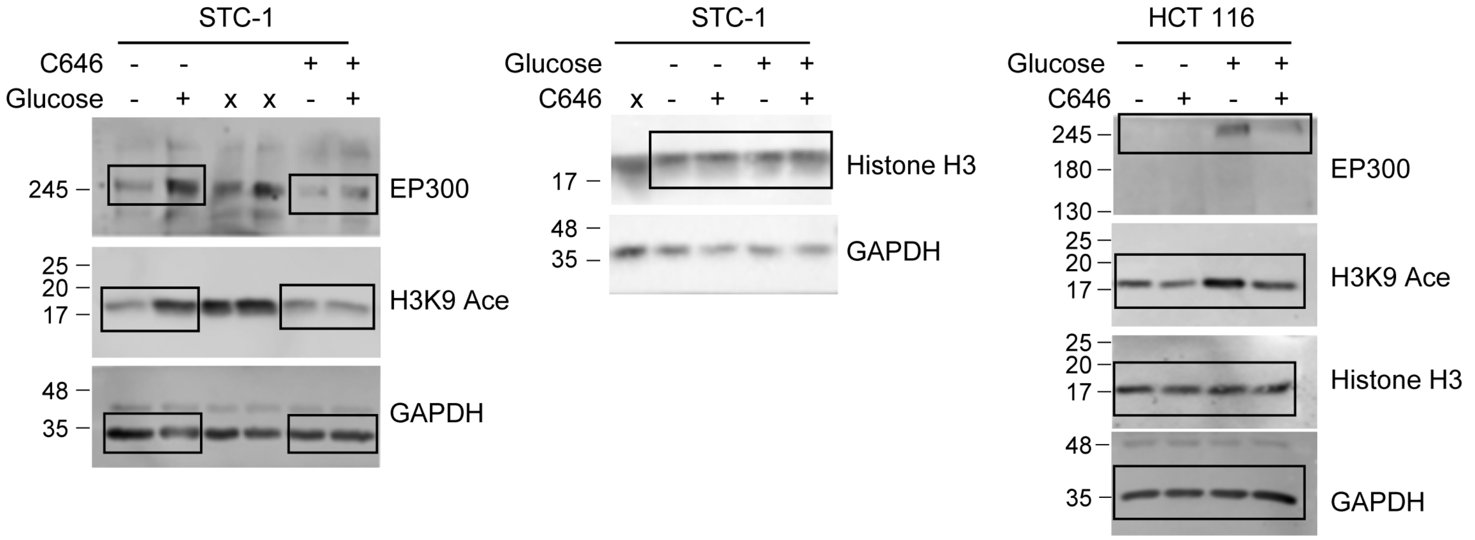

Related to Figure S2A

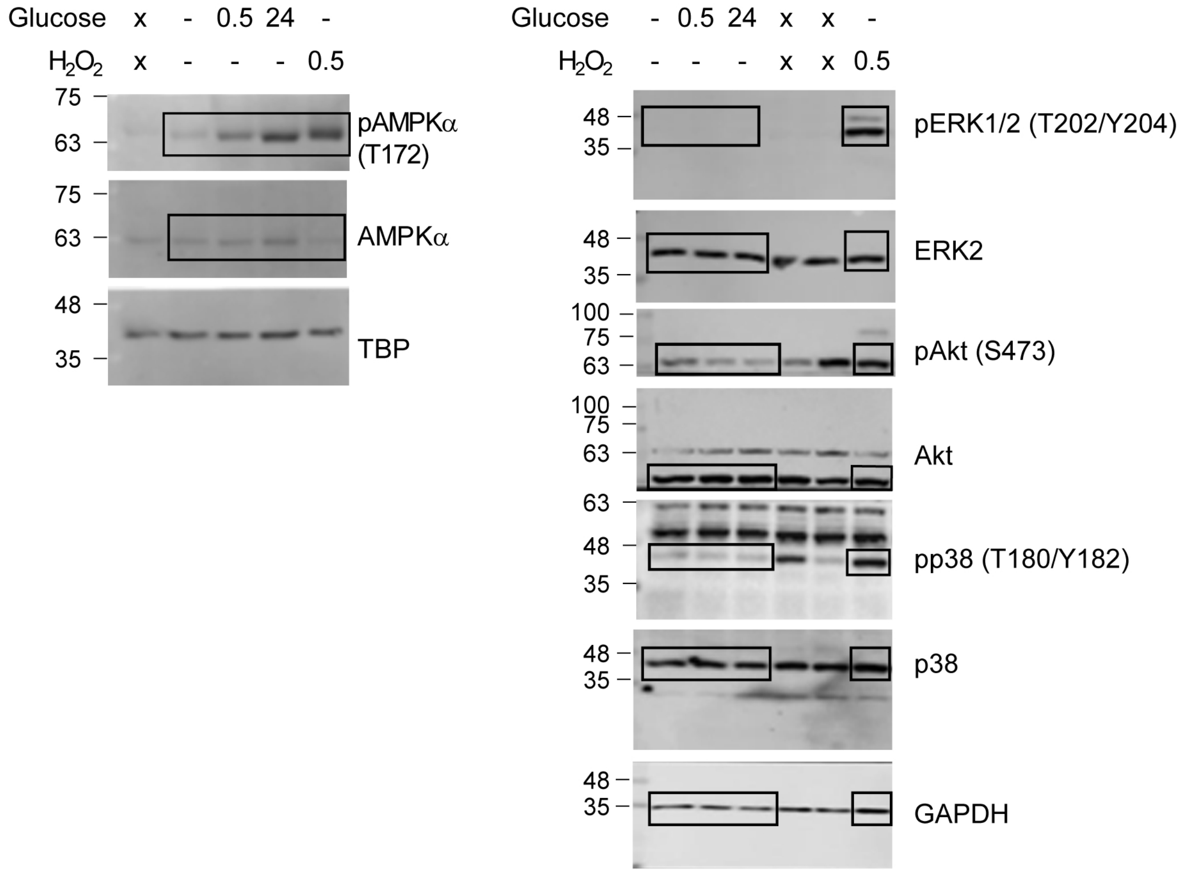

Related to Figure S3A

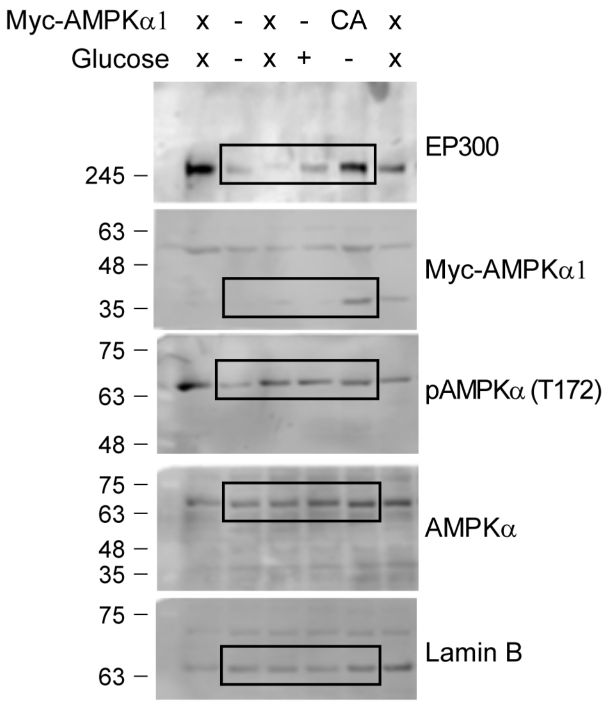

Related to Figure S3B

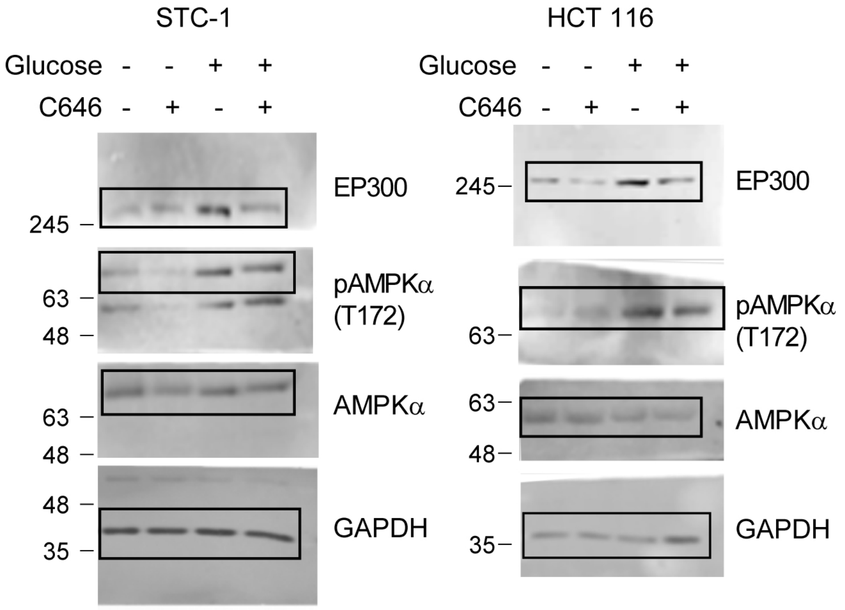

Related to Figure S3C

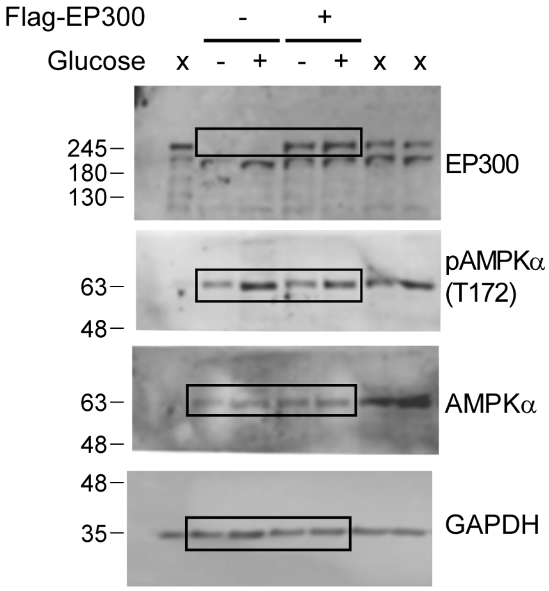

Related to Figure S4A

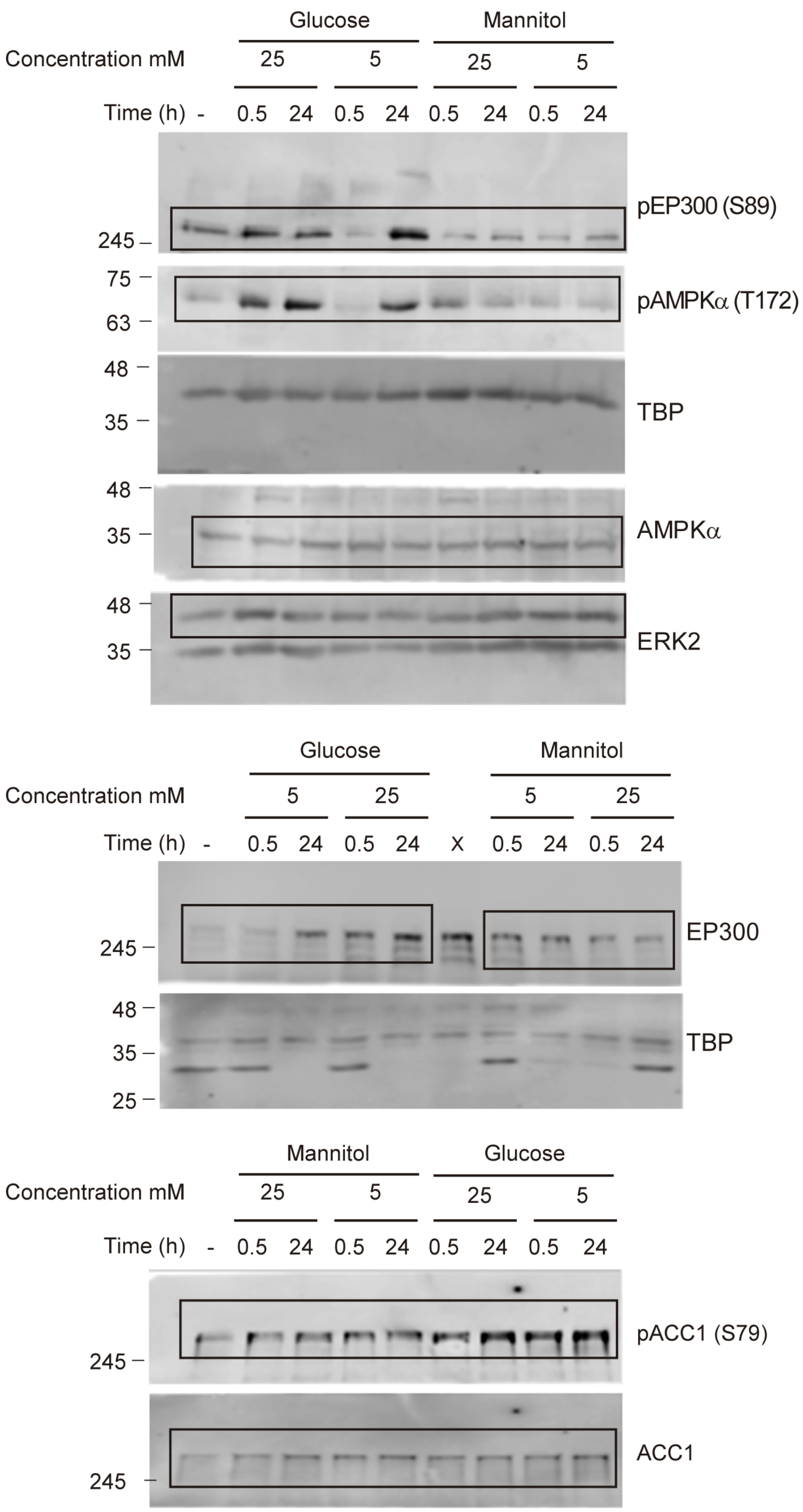

Related to Figure S4B

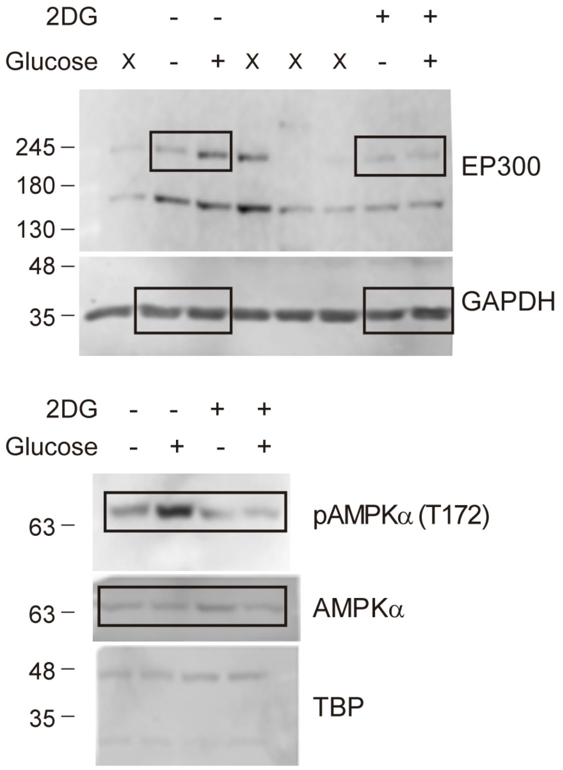

Related to Figure S4E

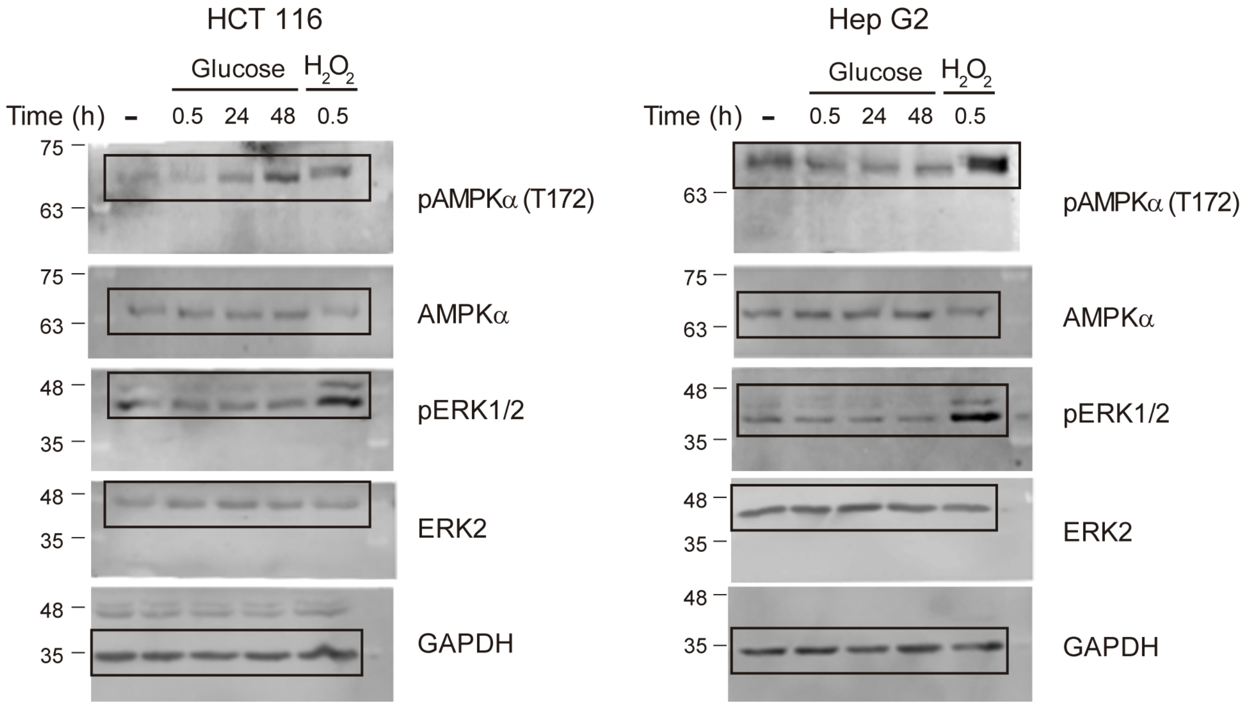

Related to Figure S4H

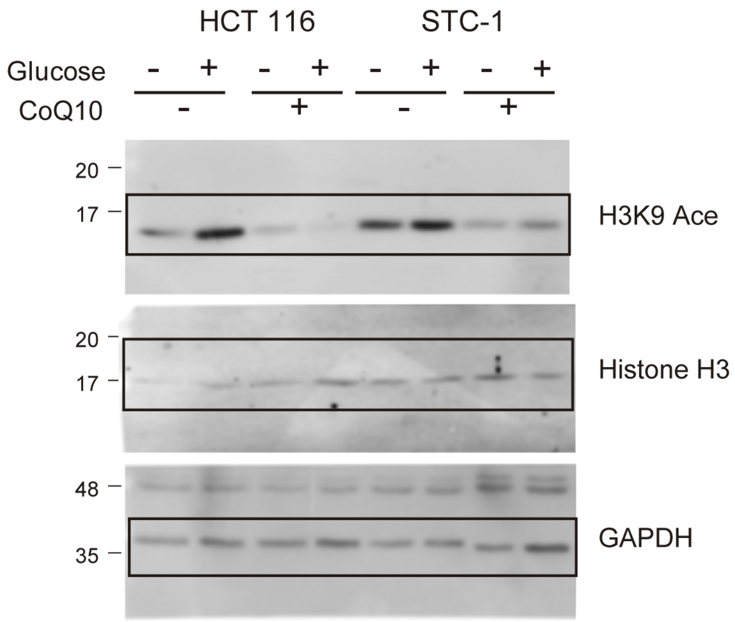

Supplement: S1 Raw Images — (PDF) [file pbio.3000732.s009.pdf]
